# Continent‐wide parallel urban evolution of increased heat tolerance in a common moth

**DOI:** 10.1111/eva.13636

**Published:** 2023-12-26

**Authors:** Thomas Merckx, Matthew E. Nielsen, Tuomas Kankaanpää, Tomáš Kadlec, Mahtab Yazdanian, Sami M. Kivelä

**Affiliations:** ^1^ WILD, Biology Department Vrije Universiteit Brussel Brussels Belgium; ^2^ Ecology and Genetics Research Unit University of Oulu Oulu Finland; ^3^ Faculty 2 Biology/Chemistry University of Bremen Bremen Germany; ^4^ Department of Ecology Czech University of Life Sciences Prague Prague Czech Republic

**Keywords:** heat knock‐down time (HKDT), heat tolerance, latitudinal variation, lepidoptera, urban evolution, urban‐heat‐island effect (UHI)

## Abstract

Urbanization and its urban‐heat‐island effect (UHI) have expanding footprints worldwide. The UHI means that urban habitats experience a higher mean and more frequent extreme high temperatures than rural habitats, impacting the ontogeny and resilience of urban biodiversity. However, many organisms occupy different microhabitats during different life stages and thus may experience the UHI differently across their development. While evolutionary changes in heat tolerance in line with the UHI have been demonstrated, it is unknown whether such evolutionary responses can vary across development. Here, using common‐garden‐reared *Chiasmia clathrata* moths from urban and rural populations from three European countries, we tested for urban evolution of heat shock tolerance in two life stages: larvae and adults. Our results indicate widespread urban evolution of increased heat tolerance in the adult stage only, suggesting that the UHI may be a stronger selective agent in adults. We also found that the difference in heat tolerance between urban and rural populations was similar to the difference between Mid‐ and North‐European regions, suggesting similarity between adaptation to the UHI and natural, latitudinal temperature variation. Our observations incentivize further research to quantify the impact of these UHI adaptations on fitness during urbanization and climate change, and to check whether life‐stage‐specific adaptations in heat tolerance are typical of other ectothermic species that manage to survive in urbanized settings.

## 1 INTRODUCTION

Rapid and global expansion of urban land cover constitutes a major threat to biodiversity because urbanization often homogenizes species communities through ecological filtering of species with exaptations to novel urban resources and stressors (Gao & O'Neill, 2020; Knop, 2016; Merckx et al., 2018). One such stressor is the urban‐heat‐island effect (UHI), with generally higher air and surface temperatures exhibited in urban areas than in surrounding rural areas because of urban–rural differences in factors such as heat retention, evapotranspiration, and convection (Manoli et al., 2019; Oke et al., 2017). As such, the UHI poses a major challenge to species in urban environments, for instance, driving community shifts towards thermophilic and heat‐tolerant species of bees, carabids, butterflies, and moths (Hamblin et al., 2017; Merckx & Van Dyck, 2019; Piano et al., 2017).

Adaptive evolutionary change is now recognized as instrumental in helping species persist in these urban settings (Diamond et al., 2022; Lambert et al., 2021; Szulkin et al., 2020). Evidence for UHI adaptation in physiological heat and cold tolerance exists in several ectothermic taxa (Diamond & Martin, 2021), including ants (Angilletta et al., 2007; Diamond et al., 2018; Martin et al., 2019), isopods (Yilmaz et al., 2021), water fleas (Brans et al., 2017) and lizards (Campbell‐Staton et al., 2020). Such evidence is essential to determine whether populations will persist or perish in response to the synergistic effects of urban heat and heat waves (He et al., 2021; Wei et al., 2022). Indeed, the ability to withstand increases in ambient temperature has proven to be a key trait in forecasting population persistence under global warming conditions (Diamond & Martin, 2021; Sinervo et al., 2010).

However, previous evolutionary studies have focused on only a single life stage, whereas organisms with multiple life stages often experience dramatically different climates and/or microclimates, especially when life stages are associated with contrasting habitats or microhabitats, leading to varied heat tolerance across multiple life stages (Medina‐Báez et al., 2023; Mutamiswa et al., 2019). Even within a single life stage, many insect larvae grow several orders of magnitude in body size, profoundly changing how they interact with their thermal environment and potentially leading to substantial changes in body temperature (Woods, 2013). Additionally, larvae typically have a distinct and narrow range of preferred body temperatures for their main activity—feeding—which can differ from the preferred body temperature range for adult activities. For instance, *Colias* spp. butterflies are characterized by maximum activity thermal requirements that differ by 10–15°C between larvae and adults (Sherman & Watt, 1973). Major differences among stage‐specific microclimates and microhabitats are likely to be general features for insects and other organisms with complex life cycles and substantial changes in body size during development (Woods, 2013). Such differences should not only occur in natural environments but also in urban settings. Here, we investigate whether different life stages show different urban evolution of thermal tolerance.

Insects are an informative model to investigate life‐stage‐specific evolution of thermal tolerance because insect populations can respond rapidly to environmental change, and their various life stages typically have different and precise ecological requirements (Hill et al., 2021; Nadeau et al., 2017). Our study species—the Latticed heath (*Chiasmia clathrata*)—is a common and widespread geometrid moth. Although its abundance has declined within the northwestern part of its palearctic range (and potentially more broadly) (Hällfors et al., 2021; Randle et al., 2019), it is typically present within cities if habitat patches contain sufficient hostplants, such as vetchlings (*Lathyrus* spp.) and vetches (*Vicia* spp.). Merckx et al. (2021) already demonstrated that urban populations of *C. clathrata* evolved seasonal developmental plasticity to take advantage of the longer growing seasons and higher temperatures associated with the UHI, but it is unknown how the potential heat stress created by the UHI impacts this species.

Here, we investigated whether the thermal physiology of *C. clathrata* has evolved in response to the UHI and whether this evolution differs between the larval and adult stages. Specifically, we contrasted the heat tolerance of individuals descended from multiple urban and rural populations, all reared in a common‐garden set‐up. Given that the UHI is associated with more frequent heat extremes (Founda & Santamouris, 2017; Zhao et al., 2018), we predicted that both larvae and adults with urban origins would tolerate heat for longer than those with rural origins. Similarly, given higher mean ambient temperatures at lower latitudes, we expected both larvae and adults from Mid‐European locations to show increased heat tolerance compared to those from North‐European origin. Our study design, involving several urban–rural paired populations across Europe, allowed us to test for repeated, parallel evolution and hence widespread urban adaptation in thermal physiology as a response to the UHI.

## 2 MATERIALS AND METHODS

### 2.1 Collecting and rearing moths

**FIGURE 1:**
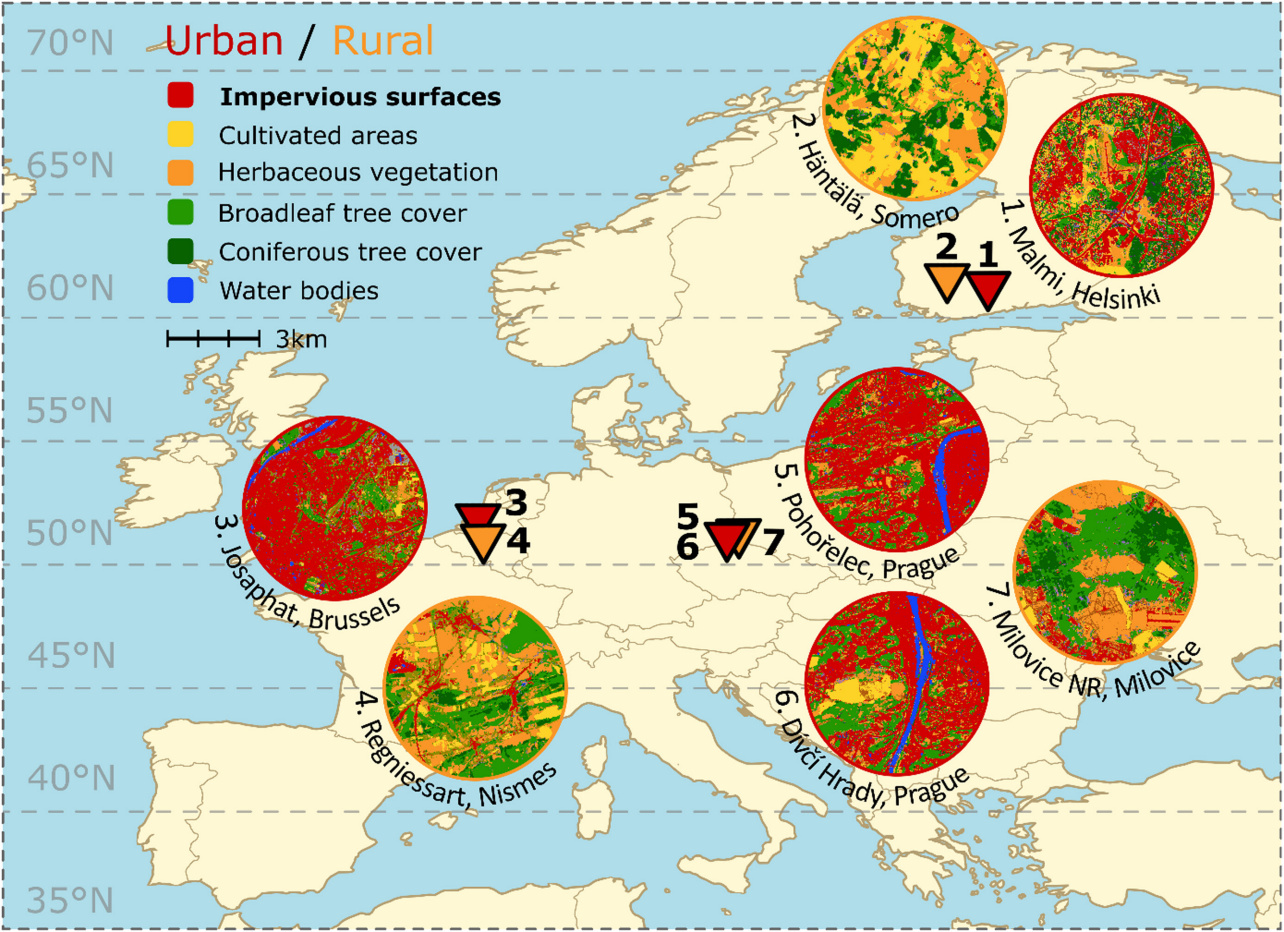
Map of Europe showing the locations of three urban (red triangles), three rural (orange triangles) sampled populations, as well as one semi‐urban (red triangle; site 6) sampled population. For each sampling site 3 km radius maps of land cover classes (Malinowski et al., 2020) are visualized. *Note*: map lines delineate study areas and do not necessarily depict accepted national boundaries.

We measured heat knock‐down times (HKDTs) from F2 and F3 generation individuals (second and third generation offspring from the parental (P) generation, respectively). Female *C. clathrata* from the P generation were individually netted in 2019 at both urban and rural sites of two Mid‐European countries (Belgium and the Czech Republic), and one North‐European country (Finland; Figure 1; Table S1). All females were taken to the University of Oulu, where they were kept at 21°C under 12L:12D and 65% relative humidity conditions (climate rooms; Arctest Oy) for egg laying in individual pots with access to sugar water. F1 and F2 generations were maintained in the laboratory with the following procedure. Newly hatched larvae were moved individually using a fine paint brush to cups (0.25 L translucent plastic). Each larva had ad libitum access to fresh shoots of the natural host plant *Lathyrus pratensis* and was reared until pupation at 21°C under 12L:12D to induce diapause. A layer of garden peat was added to the cups for pupation when the larvae reached the final, fifth instar. Five days after pupation, the pupae were excavated from the peat, and placed in a 0.25 L cup where *Sphagnum* spp. moss was added to maintain humidity. After 10 months of overwintering in a dark refrigerator room at 5°C, the pupae were moved to 20°C and 18L:6D. Eclosed females were placed with a non‐sibling male from the same population in 0.25 L cups under 21°C and 18L:6D with access to sugar water absorbed in cotton and allowed to mate and lay eggs. We measured HKDT for 54 unmated F2 adults in July 2021 after overwintering. Most individuals (634) measured for HKDT belonged to the F3 generation, which hatched in 2021 too, first the Mid‐European individuals between June 2 and 5, and then the North‐European individuals between July 11 and 14. We implemented this difference in hatching time between Mid‐ and North‐European individuals merely for practical reasons; we did not have enough space or time to process them all at once.

We raised 227 F3 individuals under 24L:0D light conditions to maximize direct development. Of them, 163 were tested as larvae (see below) and placed back in the rearing rooms immediately afterward, with the subset that managed to metamorphose (*N* = 81) to be tested again as adults (the statistical model accounted for a potential effect of being tested as a larva: see below). The other 64 were only tested as adults. We also used directly developing adults from other experiments to maximize sample size. These additional adults were raised under several different light conditions: 20L:4D (166 individuals), 20.5L:3.5D (113 individuals), and 16L:8D (128 individuals; the latter two photoperiods with and without simulated weak light pollution [Merckx et al., 2023]). The temperature was always 21°C (a standard laboratory rearing temperature: see, for example, Merckx et al., 2021, 2023). Around 5 days after pupation, pupae were excavated from the peat and sexed based on sex‐specific genital scars. After pupation, pupae were monitored daily for eclosion for a minimum of 2 weeks. Adult HKDTs were assessed 0–11 days (mean = 1.5 days; median = 1 day) after adult eclosion.

### 2.2 Heat knock‐down time (HKDT) measurements

From a total of 50 different F2 and F3 full‐sib families, 688 individuals were tested. Of these, 82 were tested in the larval stage only, 81 were tested as larvae and adults, and 525 individuals were tested in the adult stage only, resulting in 769 heat knock‐down measurements overall (Table S1). We measured HKDTs using a static assay (e.g., Angilletta et al., 2007; Nyamukondiwa et al., 2018), a common measure of heat tolerance that correlates strongly with HKDTs measured using a dynamic assay and with other heat tolerance measures, such as critical thermal maximum (CT_max_) (Berrigan, 2000; Jørgensen et al., 2019). All larvae from the subset of individuals assessed in the larval stage (*N* = 163) were tested 2 days after entering the final larval instar. For increased precision, each individual was tested separately. Each of them was weighed (Precisa 202A; precision: 0.1 mg) immediately prior to the measurement of the HKDT. After weighing, individuals were placed in a transparent plastic conical centrifuge tube (Falcon, 50 mL) without lid. This tube was then immersed until ca. 1 cm under the top of the tube in a programmable water bath (Grant Instruments Inc., Cambridge, UK: GD100), kept at 50.0°C for the duration of the experiment. A similar temperature was used in HKDT experiments with other moth species (e.g., 49–50°C: Mutamiswa et al., 2018, 2019; Tarusikirwa et al., 2020). Although 50°C may appear high, the goal of HKDT experiments is to use a high temperature as an acute heat shock, producing a measure related to CT_max_ useful for quantifying relative differences between populations. Also, note that the inside of the tube took time to reach the maximum temperature of 50°C (Figure S1). Lower temperatures would risk being below CT_max_, leading to extremely long HKDTs or no knock‐down at all.

The tube was held in place vertically by manually pressing it down to the bottom of the water bath. The HKDT (in seconds) was recorded with a timer from tube insertion into the bath until knock‐down, which was defined as when the main part of the larval body (i.e., the abdomen) stopped moving for a full 20 s (these 20 s were not included in the HKDT). All HKDT measurements and visual observations were done by one observer only (i.e., Thomas Merckx). The observer stood next to the water bath, which was on a table, and continuously looked down the tube from above, easily observing all movements of individually tested larvae and guaranteeing a high accuracy (ca. 1 s) in recorded HKDTs. During the heat exposure, larvae initially remained curled on the bottom for some seconds or immediately started crawling around, but all showed energetic crawling movements for some time before typically and suddenly being knocked down by falling motionless on their back. Immediately afterward, larvae were placed back in their individual rearing cups and climate room, with identical rearing conditions for tested and untested larvae. The tested larvae that successfully completed pupation were retested as adults (*N* = 81; i.e., 50% survival).

Adults (*N* = 606) followed a similar test procedure, except that they were not weighed and that after placing them in the test tube—now with cotton pushed into the conical tip to form a flat surface on which the adult could stand—a transparent lid covered the tube to prevent escape. We recorded HKDT as the time in seconds from tube insertion into the bath until the individual fell on its side, without being able to recover a stable position for at least 20 s (small, trembling movements of the appendages were allowed). After insertion in the tube, individuals typically waited about 20–50 s before walking and flying around until they were knocked down.

To assess the temperature range experienced by individuals during the trials, we measured the temperature trajectory inside the tube using a type K thermocouple (Mastech MS8217) to record the temperature every 10 s from insertion in the water bath until 240 s had passed. These temperature measurements occurred in separate trials without any larvae or adults in the tubes but otherwise replicated the experimental conditions (separately testing the effects of the cotton and lid). During separate trials, temperatures were measured in three distinct positions in the tube: air temperature (center of tube at the 20 mL mark), surface temperature (wall of tube at 20 mL mark), and bottom temperature (either the temperature of the cotton surface or the bottom surface without cotton). We repeated the measurements six times for each position and experimental set‐up, for 36 total measurement sequences. Although we acknowledge that temperature readings may differ with and without an insect individual within the tube, we expect such differences to be minimal.

During the trial, air and bottom temperatures initially rose rapidly for the first 70–100 s, followed by gradual deceleration of the temperature increase until equilibrium after approximately 200 s (Figure S1). The wall temperature reached equilibrium faster, approximately after 150 s (Figure S1). As expected, air temperatures were cooler and slower to heat than surface temperatures, but both followed similar trajectories across experimental set‐ups. Most heat knock‐downs occurred between 60 and 150 s. During this time, mean temperatures increased at a rate of ca. 0.03–0.2°C/s and ranged from 41.7 to 48.1°C for air temperature and 38.4–49.4°C for surface temperature. The temperature at the bottom of the tube was quite different with and without the cotton but fell broadly within the range of air and surface temperatures: the cotton surface followed a trajectory similar to the air temperature, whereas the plastic bottom followed a trajectory similar to the wall temperature. Therefore, both life stages were exposed to a similar range of temperatures, especially since adults engaged in flight within the tube, but the cooler bottom temperature for adults means that the same HKDT may nonetheless reflect a slightly cooler temperature for them.

### 2.3 Statistical analyses

Variation in larval HKDT was analyzed with linear mixed‐effects models fitted with the maximum likelihood method using the function “lme” (package “nlme”; Pinheiro et al., 2021) in R version 4.1.2 (R Core Team, 2021). Larval HKDT was the response variable and mass, environment (rural/urban), and their interaction as well as region (Mid‐European/North‐European) were used as fixed effects. For the mass variable, we used centered mass (i.e., mass subtracted by mean mass). We used family (i.e., parental lineage) as a random effect for intercepts. Residual variance was higher in Mid‐European than in North‐European populations, so this heteroscedasticity was modeled by adding weights using the “varIdent” function (Pinheiro et al., 2021) so that different residual variances were allowed in the two regions.

We derived all meaningful submodels from the (global) model described above with the “dredge” function (package “MuMIn”; Barton, 2020), and then compared the models on the basis of AICc (Akaike Information Criterion corrected for small sample size). To avoid overconfident conclusions concerning the factors of interest, we used model averaging instead of model selection (see, e.g., Forstmeier & Schielzeth, 2011). We averaged fixed effects across a set of models that were <8 AICc units from the best model (i.e., the model with the lowest AICc value) and were not more complex (i.e., did not include more parameters) than the best model (see also Burnham & Anderson, 2002; Richards et al., 2011) using the function “model.avg” (Barton, 2020). To derive confidence limits for model‐averaged predictions, we used the bootstrap approach. We took resamples from the data with replacement so that resampling was stratified within the rural and urban environments, then fitted the global model to these data, derived all meaningful submodels, averaged fixed effects across the models, and derived model‐averaged (full average) regression lines for HKDT variation in relation to centered mass separately for rural and urban larvae. This procedure was repeated 5000 times. Note that we averaged across all the models derived from the global model in the bootstrap approach because otherwise model averaging failed with some bootstrap realizations of the data. Also, we did not take the region variable into account in the bootstrap analysis, because it did not explain variation in HKDTs (see Results). Then, we derived 95% percentile confidence intervals (i.e., excluded the extreme low and high 2.5% from the bootstrap distributions) for the environment‐specific regressions and used the means of bootstrap distributions as the point estimates.

Because adults measured for HKDT originated from different rearing conditions and generations, we first checked if HKDT depended on these factors. Visual data evaluation and formal analysis did not indicate any interactions between rearing conditions and other variables (see Table S2) and thus exclude the possibility that results are confounded by rearing origin. Therefore, to avoid overfitting and problems in estimating all model parameters (see below), we did not take rearing origin into account in the main analysis of adult HKDT. We analyzed variation in adult HKDT by using the same approach as described above for larval HKDT, except that the fixed effects included age since eclosion (days; continuous), environment (rural/urban), sex, and all interactions among these three variables, as well as region (Mid‐European/North‐European), its interaction with environment, and an indicator variable for whether the same individual had also been measured as a larva (no/yes). The model was heteroskedastic as the residual variance differed between regions and sexes, so we added weights by the variance function “varIdent” specifying different residual variances for each combination of region and sex (Pinheiro et al., 2021). Adding this heteroscedasticity modeling reduced the AICc value of the model by 33.9 units. Because family‐level variance could not be estimated accurately, we further simplified the model by removing the interaction between region and environment, because there was no evidence of such an interaction. Only the main effect of region was retained. This model simplification slightly improved the accuracy of the family‐level variance estimation. Deriving from this “global” model, we got 76 models, 11 of which were <8 AICc units from the best model, and two of them were not more complex than the best model (cf. Burnham & Anderson, 2002; Richards et al., 2011). We averaged the fixed effects across these two models and derived confidence intervals for the model‐averaged regressions similarly as explained above, but bootstrap resampling was now stratified within the combinations of environment, region, and sex. We evaluated the sensitivity of the results to the presence of the semi‐urban Dívčí Hrady population by repeating the analyses mentioned above without the Dívčí Hrady population.

We also assessed whether larval and adult HKDTs are correlated by calculating Pearson's correlation between family‐specific mean larval and adult HKDTs using only individuals measured as both larvae and adults. We estimated heat knock‐down temperature by rounding the HKDT to the nearest 10 s and using our corresponding estimate of the mean bottom temperature at that time.

## 3 RESULTS

**FIGURE 2:**
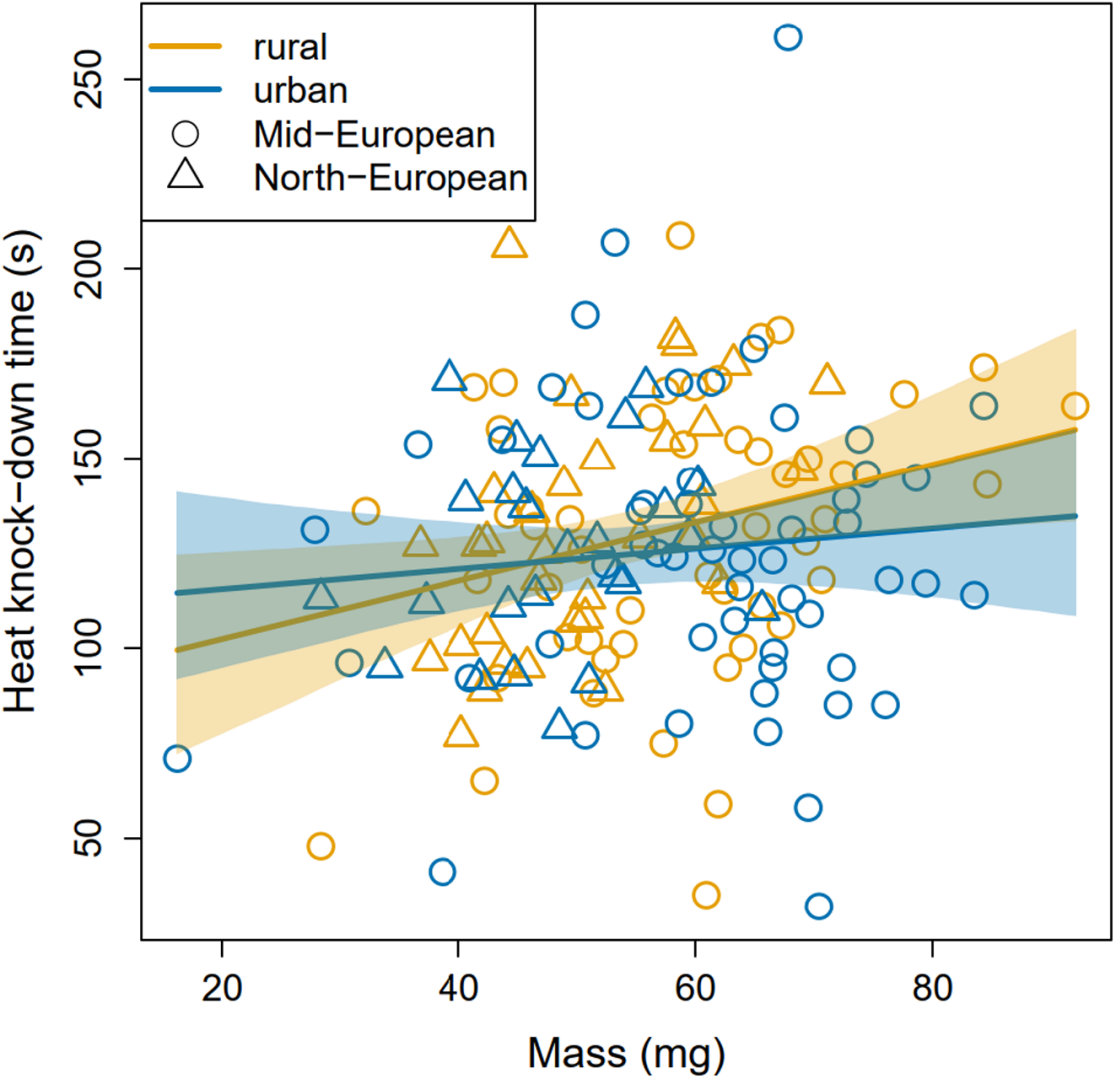
Larval heat knock‐down time in relation to mass at measurement in both urban (blue symbols and lines) and rural (yellow symbols and lines) populations from Mid‐Europe (circles) and North‐Europe (triangles). The shaded areas around the regression lines show 95% bootstrap confidence intervals.

**FIGURE 3:**
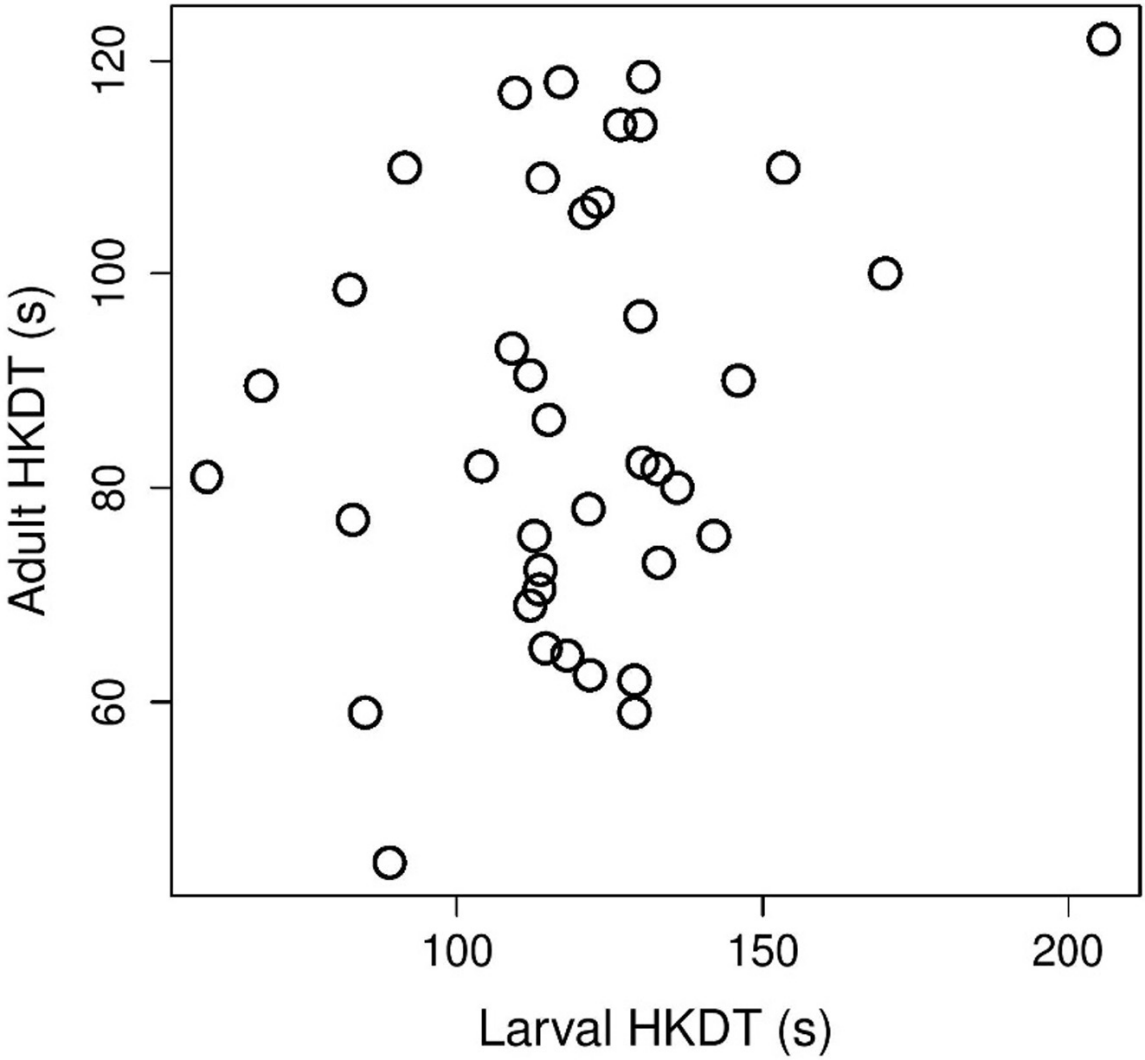
Scatterplot of family means showing the weak and only marginally significant correlation between larval and adult heat knock‐down time (HKDT).

Larval HKDT ranged from 32 to 261 s (mean = 127 s, SD = 35.4 s); while there was no significant difference between urban and rural individuals (see the “environment” effect in Table 1), HKDT increased slightly but significantly with increasing larval body mass (Table 1; Table S3; Figure 2). Analysis without the semi‐urban Dívčí Hrady population led to essentially identical results (Table S4). For individuals measured at both stages, larval and adult HKDT were marginally correlated (*r* = 0.303, *t* = 1.935, df = 37, *p* = 0.061; Figure 3), but the estimated temperatures at which the larvae were knocked down tended to be higher than the adult knock‐down temperatures (mean [95% CI] larval temperature = 49.0 [48.8, 49.2]°C; mean adult temperature = 43.6 [42.8, 44.4]°C).

**TABLE 1:** Model‐averaged fixed effects (full average) of linear mixed‐effects models explaining heat knock‐down time (s) in larval *C. clathrata* in relation to environment of origin (rural/urban), centered body mass (mg) at time of testing (Centered mass) and region (North‐European/Mid‐European).

Model parameter	Estimate	Adjusted SE	*Z* value	*p* value
Intercept	129	5.12	25.2	<0.0001
Environment (urban)	−4.11	5.70	0.720	0.47
Centered mass	0.877	0.354	2.47	0.013
Region (North‐European)	2.70	5.38	0.501	0.62
Environment (urban) × Centered mass	−0.396	0.542	0.731	0.46

*Note*: The set of models included in averaging is presented in Table S3.

**FIGURE 4:**
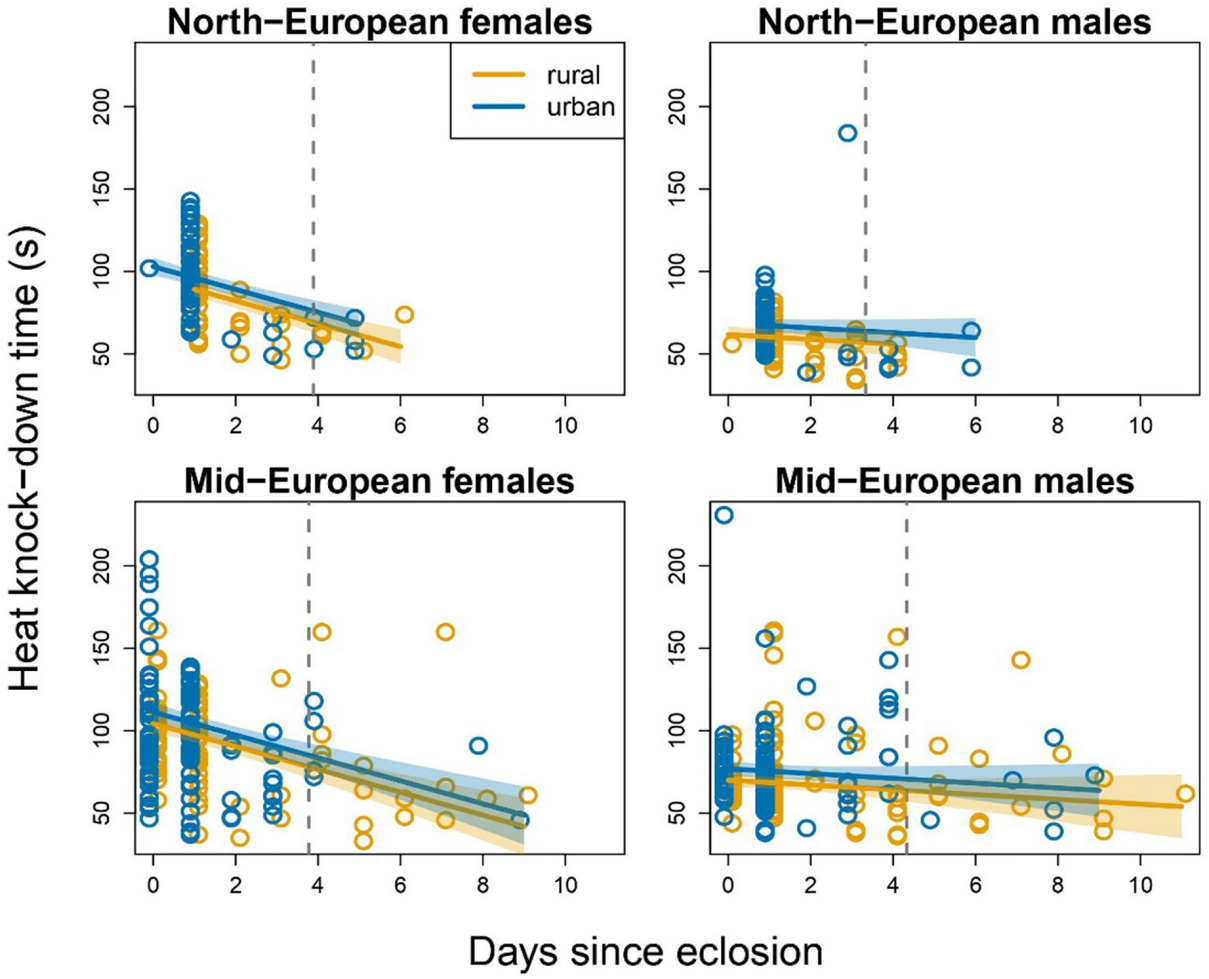
Heat knock‐down time (HKDT) (s) in *C. clathrata* adult females (left column) and males (right column) from North‐European (top row) and Mid‐European (bottom row) populations (*N* = 606) in relation to age (days) since eclosion. Each panel shows the original measurements for individuals deriving from rural (orange symbols) and urban (blue symbols) populations. Note that the age variable has only integer values but that data points are slightly jittered along the horizontal axis for visualization. The regression lines (orange and blue lines for rural and urban populations, respectively) and their 95% percentile confidence intervals (shaded areas around the lines) are derived from 5000 model‐averaged estimates based on bootstrap resampling. The gray vertical dashed lines indicate the age before which the fitted regression line for the urban population falls outside of the confidence interval of the regression for the rural population; that is, there is statistical support for a longer HKDT in the urban than in the rural population below that age (note that the confidence intervals are narrow at young ages as most data are concentrated in the youngest age classes) (see also Figure 5).

**FIGURE 5:**
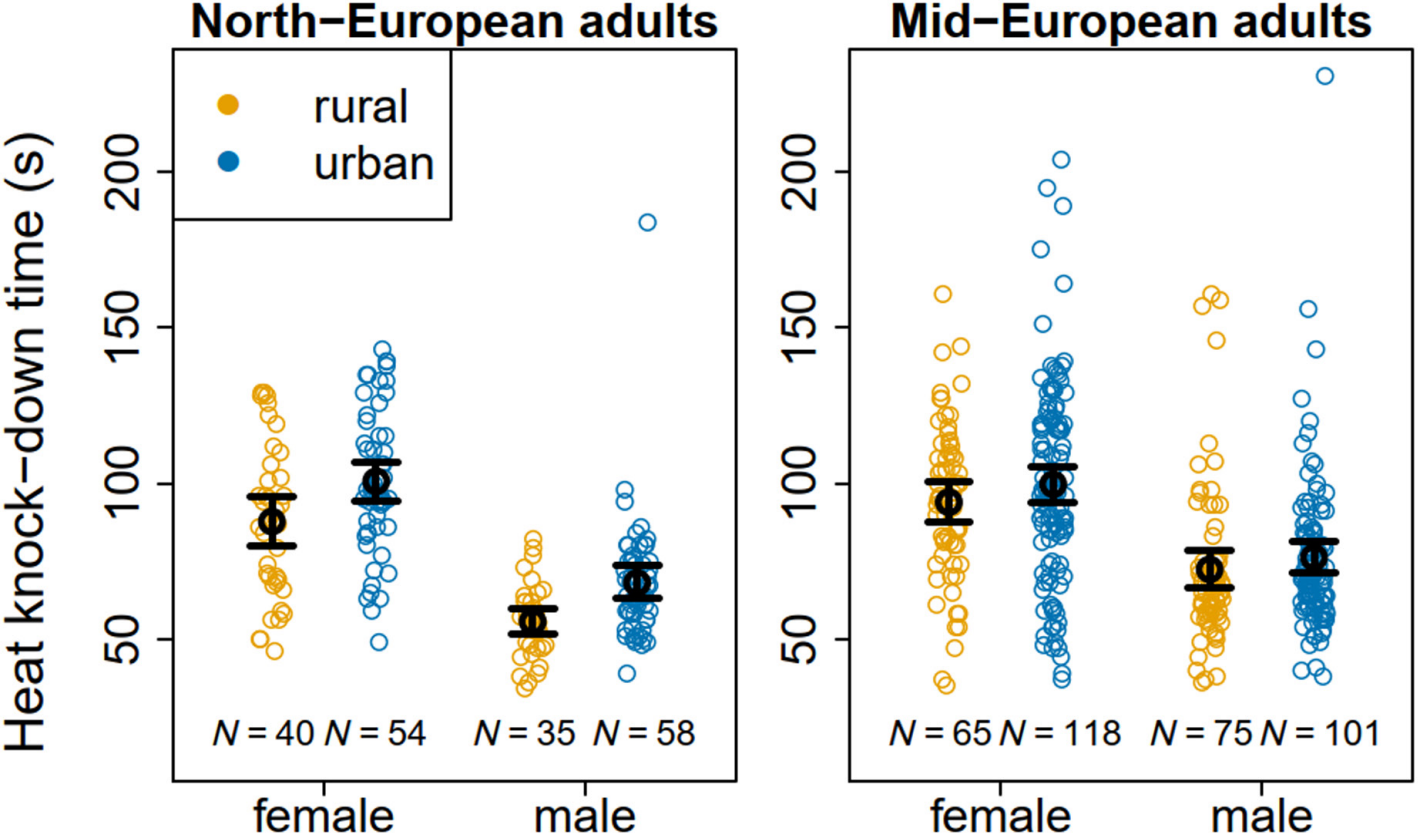
Raw data (open circles) on heat knock‐down time (s) in young *C. clathrata* adults (Mid‐European males: ≤4 days since eclosion; all other groups: ≤3 days since eclosion; i.e., all data left to the vertical dashed lines in Figure 4). Data are shown for females (left) and males (right) from urban (blue) and rural (orange) populations from North‐European (left panel) and Mid‐European (right panel) populations (*N*_total_ = 546). Black circles show group‐specific means and black whiskers around them the 95% confidence intervals of the means. Group‐specific sample sizes are indicated at the bottom of the figure.

In adults, HKDT ranged from 33 to 231 s (mean = 82.3 s, SD = 29.4 s). At age 0 days, females from urban populations had 8.9% and 14% longer HKDTs (i.e., ca. 7 s) than those from rural populations in Mid‐ and North‐European populations, respectively (Table 2; Figures 4 and 5) and, since there was no environment by sex interaction, the difference is similar in males (Table 2; Figures 4 and 5; relative difference 5.1% and 21% in Mid‐ and North‐European populations, respectively). Young females tolerated heat longer than males (Figure 5), but female HKDT declined with age, whereas male HKDT was practically independent of age, which is why the between‐sex difference in HKDT decreased with increasing age (Figure 4), resulting in a significant age‐by‐sex interaction (Table 2; Table S5). Individuals from Mid‐European populations tolerated heat longer than those from North‐European populations, the difference in HKDT being ca. 9.9% (i.e., ca. 8 s) (at age 0 days) (Table 2; Table S5; Figures 4 and 5). These results remained basically the same when the analysis was repeated without the semi‐urban Dívčí Hrady population (Table S6; Figure S2).

**TABLE 2:** Model‐averaged fixed effects (full average) of linear mixed‐effects models explaining variation in heat knock‐down time (s) in adult *C. clathrata* in relation to age since adult eclosion (days), environment of origin (rural/urban), region (North‐European/Mid‐European) and sex.

Model parameter	Estimate	Adjusted SE	*Z* value	*p* value
Intercept	103	2.73	37.7	<0.0001
Age	−6.63	0.995	6.66	<0.0001
Environment (urban)	6.77	2.44	2.77	0.0056
Region (North‐European)	−7.67	2.19	3.51	0.00046
Sex (male)	−33.7	2.68	12.6	<0.0001
Age × Sex (male)	5.73	1.23	4.65	<0.0001

*Note*: The set of models included in averaging is presented in Table S5.

## 4 DISCUSSION

Our common‐garden experiment using individuals descended from multiple urban and rural populations across Europe demonstrated clear evidence for repeated urban evolution of ca. 10% increased heat tolerance in a geometrid moth, but only in the adult stage and not in the larval stage. The evolution of increased heat tolerance in urban populations is consistent with earlier experimental work on insects; however, previous studies have only considered single life stages, and they also have not considered flying life stages (Diamond et al., 2018; Martin et al., 2019). Urban–rural comparisons from a slightly broader diversity of insects come from field studies that also show a general pattern of increased heat tolerance in urban populations (reviewed by Diamond & Martin, 2021).

Although European cities are typically older than the North‐American cities considered in these previous studies, they have only expanded substantially since the 19th century. Because the UHI is closely related to city or population size (Manoli et al., 2019)—and likely other features of modern urban development—strong selection by the UHI should also be relatively new in Europe, and thus our study likely represents another example of rapid urban evolution. Our study suggests contrasting urban evolution across development because we found evidence for urban evolution of heat tolerance only in the adult stage and not in the larval stage of *C. clathrata*. Hence, urban adaptation in a given life stage cannot automatically be assumed to be present in other life stages too (see also Loeschke & Krebs, 1996; Bowler & Terblanche, 2008; van Heerwaarden et al., 2012 for life‐stage‐specific thermal adaptations in non‐urban contexts). Because different life stages are ecologically distinct and frequently occupy contrasting microhabitats (Kingsolver et al., 2011), UHI likely inflicts different selection on the thermal physiology of larvae and adults.

The higher (ca. 54%) overall heat tolerance in larvae compared to adults observed in the present study could be a factor explaining the absence of urban evolution in heat tolerance in the larval stage, and the presence of it in the adult stage in our study species. Because larvae are less mobile than winged adults, larval behavioral thermoregulation may be more constrained, and caterpillars of grassland species may hence have been forced to naturally evolve higher heat tolerance than adults (see also Moghadam et al. (2019), for a similar reasoning regarding *Drosophila melanogaster*). This, in combination with some scope for larval behavioral thermoregulation by moving toward lower and cooler positions within the evapotranspiration‐ and shade‐providing grass sward (Braem et al., 2023), could mean that the larval heat tolerance limit is not often reached—meaning no selection for increased heat tolerance—even in the hotter urban environments. While the scope for larval behavioral thermoregulation within the vegetation may be similar in urban and non‐urban grasslands, the scope for adult behavioral thermoregulation may be lower in urban settings because this happens at a larger spatial scale, and suitable habitat—within the hot urban matrix—is typically reduced and more fragmented in urban settings (Merckx et al., 2018). As such, for adult moths—this species is also diurnally active (Leraut, 2009)—it may be more difficult to locate sufficiently cool microsites during heat wave conditions in urban settings compared to many rural/natural settings with potentially more options for thermoregulation. This, together with their relatively low heat tolerance, may lead to adult heat tolerance limits being reached more often in urban than non‐urban settings, explaining the selection for increased adult heat tolerance in urban landscapes. In addition, since sample size of larvae was much lower than of adults (163 vs. 606, respectively), it is possible that the lack of statistically supported urban environment effects for larvae is—at least partially—a consequence of limited sample size. To assess this possibility, we resampled larval data to the adult sample size in a bootstrap re‐analysis of larval heat tolerance (Figure S3). This analysis suggested that the interaction shown in Figure 2 would become stronger and statistically supported with a larger dataset, but that this would not be the case for the main urban environment effect. Consequently, although we cannot completely exclude a possibility for small‐scale urban adaptation in larval heat tolerance, we can quite confidently exclude the possibility of substantial urban adaptations in larvae.

Because we measured laboratory‐reared F2 and F3‐generation individuals raised at a constant temperature, we can rule out transgenerational plasticity (i.e., parental effects) due to thermal conditions experienced by the preceding generations, and the differences we found should have a heritable basis. Although larval and adult heat tolerance appeared not to be entirely independent, the positive correlation was not strong (*r* = 0.3) and only marginally significant. Such a weak correlation potentially allows separate evolutionary responses at the larval versus adult stage, enabling the life‐stage‐specific evolution of thermal physiology we found. Indeed, gene expression can be life‐stage‐specific for genes responsible for resistance to stressors (e.g., Arias et al., 2011; Yang et al., 2013).

Although we did not find any variation between populations in HKDT in larvae, we still found substantial individual variation in larval HKDT, specifically associated with body mass. Heavier larvae withstood heat longer than smaller ones. This could be due to a physiological difference in heat tolerance with size, but it could also be explained simply by the physical effects of body size. The studied larvae varied about four‐fold in body mass, and larger organisms will heat more slowly than smaller ones. Although the larvae we studied should reach equilibrium temperatures quickly due to their small absolute size (<100 mg) (Stevenson, 1985), our experiment heated on the order of seconds, rapidly enough that the larger larvae may have simply had a cooler body temperature at any given time during the measurements. We were unable to measure the mass of the adults and therefore could not test for a similar effect in adults; however, we found a substantial effect of sex on adult HKDT. Female *C. clathrata* are heavier than males (e.g., Välimäki et al., 2013), and this mass difference between the sexes could explain some of the difference in HKDT with generally longer HKDT in females than in males, although sexual dimorphism is also common in stress traits independent of size (e.g., Niveditha et al., 2017). The mass difference between females and males may also explain why we only found an effect of age on HKDT for the females; both sexes lose weight with age, but females more so than males. This lost body mass—along with generally reduced vitality, strength, and resilience with increasing age—may explain why older females were knocked down sooner than younger ones.

We studied three pairs of urban/rural populations across Europe, and the relatively low mobility of the study species (Kuussaari et al., 2014) makes urban‐to‐urban dispersal unlikely. Thus, we conclude that parallel, repeated, and hence widespread urban evolution of adult heat tolerance has likely occurred in *C. clathrata* in several cities. Moreover, we show that Mid‐European adult moths are more heat tolerant than North‐European ones, as expected if lower‐latitude populations have adapted to the warmer climate at lower latitudes. This result is consistent with latitudinal variation in HKDT in *Drosophila melanogaster* (Sgrò et al., 2010), and upper thermal limits of terrestrial ectotherms, including insects, generally weakly decrease with increasing latitude (Sunday et al., 2019), although latitudinal clines in heat tolerance are complex in some species (van Heerwaarden et al., 2012). The relative difference in HKDT between urban and rural populations was approximately 10%, which mirrors the difference between the Mid‐European and North‐European regions. This result fits well with the positive association between trait changes along thermal urbanization and geographic gradients found across studies, suggesting that cities can be seen as surrogates for lower‐latitude conditions and maybe for future global warming conditions as well (Diamond & Martin, 2021; Lahr et al., 2018).

Our study was not designed to measure CT_max_. Instead, the HKDTs reported here are relative measures of heat tolerance under a short‐term heat shock when comparing different populations, regions, and sexes within our study. When comparing life stages, larvae resisted heat for longer (ca. 54%) than adults. Since hot and dry conditions are typically stressful for insects (Mutamiswa et al., 2019), we consider this a conservative result because larvae likely experienced higher temperatures and potentially slightly drier conditions than adults due to differences in the experimental set‐up for tested larvae versus adults (i.e., absence of cotton in, and no lid on top of the larval test tube, meaning no thermal insulation and less build‐up of humidity, respectively). This finding of increased heat tolerance at the larval versus the adult stage qualitatively matches results obtained in other moths from non‐urban populations with a similar methodology (Mutamiswa et al., 2018; Tarusikirwa et al., 2020). However, it remains unclear how well HKDTs, or other measures of short‐term heat shock tolerance, predict the ability to tolerate long‐term exposures to elevated temperatures in nature. Under natural conditions (including heat waves), temperature increases much more slowly than in our experiment, and acclimation—though often limited—may be possible, increasing heat tolerance (Kellermann et al., 2017; Ma et al., 2021). As such, if there was any acclimation or other plastic effects on heat tolerance in cities, we predict that the differences in the field would be even greater than the observed evolved differences. Additionally, both wild adults and larvae have the opportunity for behavioral thermoregulation by changing microhabitats to avoid elevated temperatures (Kearney et al., 2009; Tropek et al., 2017), although larvae may be limited to the microhabitats located on or near their host plants (Casey, 1976; Kaiser et al., 2016; Nielsen, 2017). Thus, further research will be required to determine if and how the differences in heat tolerance we observed in the lab manifest under natural conditions, and whether they provide an adaptive benefit in cities.

Grassland habitats are characterized by greater temperature variability and substantially greater maxima than regional conditions (Bernath‐Plaisted et al., 2023); during hot and sunny conditions they can be exposed to temperature extremes over 45°C in temperate regions (Bernath‐Plaisted et al., 2023; Gardiner & Hassall, 2009; Suggitt et al., 2018) and well above 50°C in continental regions (Carroll et al., 2016). Under global change, the frequency and intensity of high temperatures have increased and continue to increase, particularly in thermally stressful environments like grasslands and cities. This has obvious fitness consequences for grassland Lepidoptera and other insect taxa (Ma et al., 2021), especially since microclimate is far more relevant than the background climate for relatively small organisms such as insects (Braem et al., 2023; Suggitt et al., 2011). Because thermal extremes are key causes of insect responses to global change (Ma et al., 2021) and are known to induce evolution of thermal tolerance (Buckley & Huey, 2016; Hoffmann et al., 2013), we believe that the relative difference in tolerance of extreme temperatures we find here will be relevant to ecological and evolutionary processes in nature. Furthermore, our results do suggest the importance of urban–rural differences in within‐habitat microclimatic variation and indicate that variation in UHI impacts across microhabitats would be a worthwhile topic for future research.

Given the generality and biological relevance of the UHI for the fitness of ectothermic organisms, repeated evolution of thermal physiology is expected across species in many cities (Campbell‐Staton et al., 2020). We provide evidence for repeated urban evolution of increased heat tolerance in a common and widespread moth, consistent with expected heat stress caused by the UHI; however, in our study, urban evolution occurred only in the adult and not in the larval stage. Hence, urban evolution may vary across life stages, and different life stages can adapt differently to the UHI, and probably to other urban‐related stressors too. Our findings stress the need to consider how the UHI affects the whole life cycle, and how the impacts of the UHI may vary among the different microhabitats used by different life stages. Furthermore, urban environments are likely to heat further due to the synergistic effects of the UHI and climate warming (Chapman et al., 2017). Therefore, adequately designing and managing green and blue urban infrastructure will be crucial both to reduce the UHI (together with other urban stressors) and to sufficiently increase the spatial heterogeneity of microclimates and microhabitats across life stages (Pincebourde et al., 2016; Suggitt et al., 2018). Doing so may allow sufficient time for adaptive genetic responses, resulting in more effective biodiversity conservation in cities (Lambert & Donihue, 2020).

## CONFLICT OF INTEREST STATEMENT

The authors have no conflict of interest to declare.

## Supporting information


Data S1.

## Data Availability

The data that support the findings of this study are openly available in the Dryad Digital Repository at https://doi.org/10.5061/dryad.6q573n638, Merckx et al., 2024.
