# Supplementary material for: Continent‐wide parallel urban evolution of increased heat tolerance in a common moth
Source: Evol Appl. 2023 Dec 26;17(1):e13636. doi: 10.1111/eva.13636 (PMC10810253; doi:10.1111/eva.13636)
Supplement: Supplementary file 1 — Data S1. [file EVA-17-e13636-s001.docx]

**Supporting Information (Continent-wide parallel urban evolution of increased heat tolerance in a common moth)**

**HKDT methods verification**

**Fig. S1:** Temperature as a function of time since the beginning of a heat knock-down trial in air in the middle of the measurement tube (top panel), on the bottom of the tube (middle panel), and on the wall of the tube (bottom panel). Temperature change is shown for both the set up used for measuring larvae (blue symbols) and adults (black symbols). Points indicate time-specific means across six separate measurement rounds, and whiskers the 95% CIs of the means.


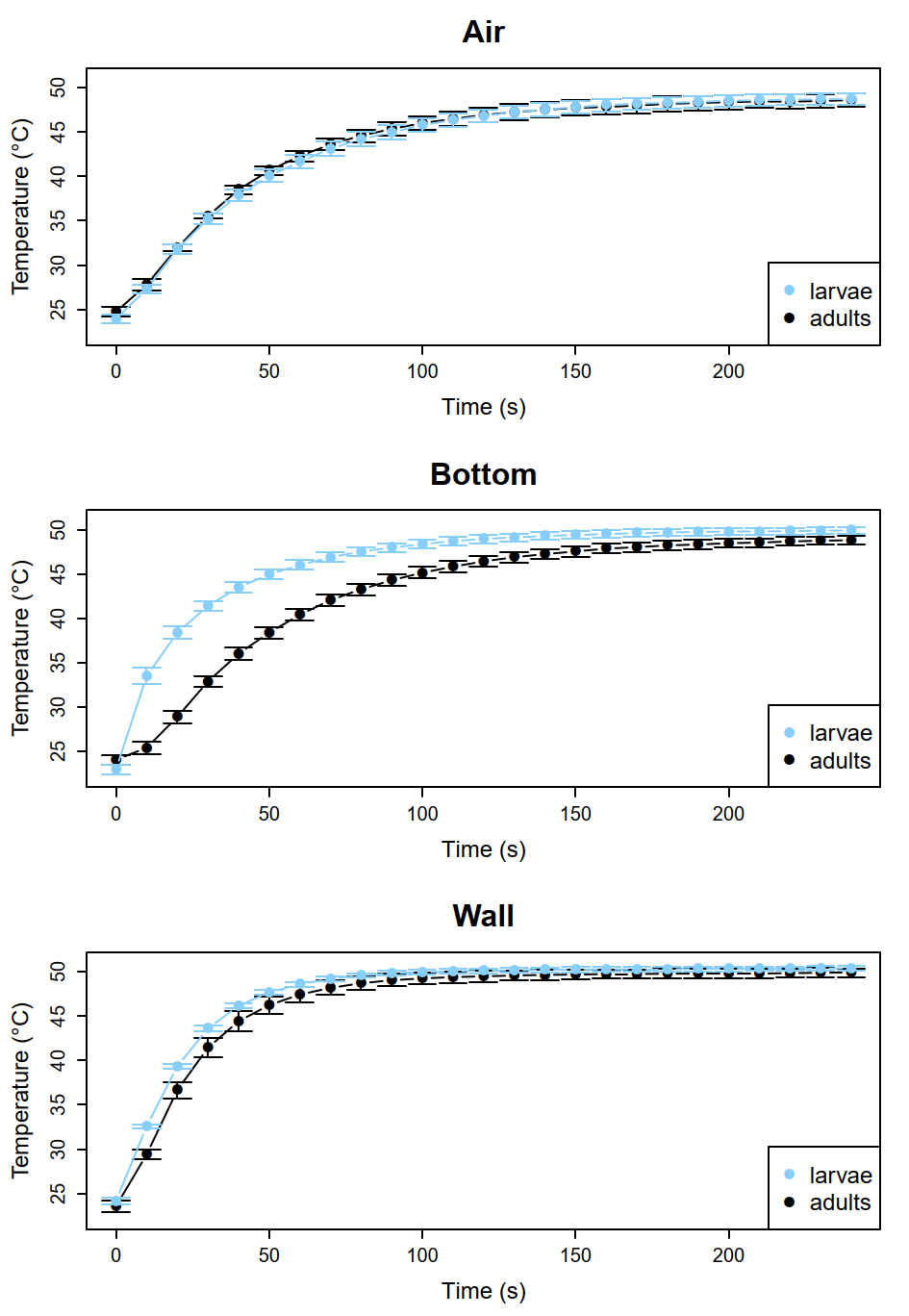


**Fig. S2:** Same as Figure 4, except excluding the semi-urban Dívčí Hrady population from the analysis. Heat knock-down time (s) in *C. clathrata* adult females (left column) and males (right column) from North-European (top row) and Mid-European (bottom row) populations (N = 525) in relation to age (days) since eclosion. Each panel shows the original measurements for individuals deriving from rural (orange symbols) and urban (blue symbols) populations. Note that the age variable has only integer values but that data points are slightly jittered along the horizontal axis for visualization. The regression lines (orange lines for rural population; blue lines for urban populations) and their 95% percentile confidence intervals (shaded areas around the lines) are derived from 5000 model-averaged estimates based on bootstrap resampling. The gray vertical dashed lines indicate the age, below which the fitted regression line for the urban population falls outside of the confidence interval of the regression for the rural population, that is, there is statistical support for a longer heat knock-down time in the urban than in the rural population below that age.


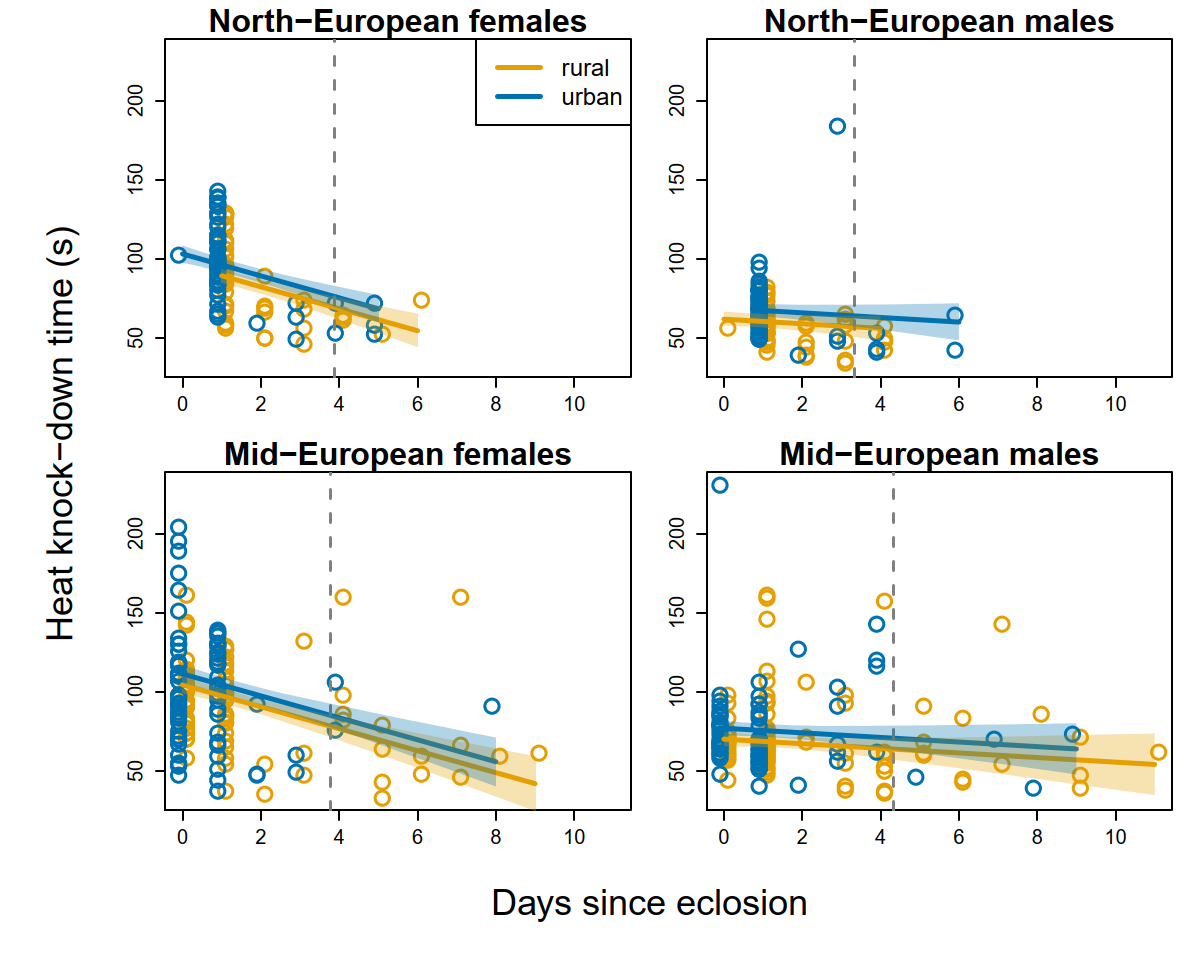


**Fig. S3:** Results of a resampling analysis for assessing whether inferences concerning larval heat tolerance would change if sample size was similar to the sample size for adults. We randomly resampled larval data with replacement so that the resulting sample size matched the adult sample size (i.e., 606 observations in total). This resampling was stratified so that we maintained the sample sizes for each combination of environment (rural / urban) and region (Mid-European / North-European) in the adult data when resampling the larval data (i.e., 169 and 231 rural and urban individuals for the Mid-European region, respectively, the corresponding numbers for the North-European region being 84 and 122). Then, we fitted the model that was the best for the original data (see Table S3) to the resampled data. This model included centered mass, environment and their interaction as fixed effects, family as a random effect, and heteroscedasticity modelling allowing different residual variances for different regions. Next, we derived all simpler models from this ‘global model’ similarly as explained in the main text, and included only models that were within 8 AICc units from the best model for further consideration. If only a single model was retained by this condition, we stored the fixed effects and their standard errors from that model, and otherwise we averaged across the set of retained models and stored the model-averaged (full average) fixed effects and their adjusted standard errors. This procedure was repeated for 5000 times. We ignored the ‘region’ variable here because it turned out that our data did not allow a reliable estimation of the effect of increased sample size on the region effect. This was evident because the inclusion of the ‘region’ variable resulted in a highly bimodal bootstrap distribution of region effect sizes, one peak being negative and the other positive, and such lability of the region effect may bias the estimates for the other fixed effects.

We compared the model-averaged fixed effects based on models fitted to the original larval data (Table 1) to the bootstrap distributions of the same effects derived with the procedure explained above (top row). The histograms illustrate the bootstrap distributions. The vertical red lines are the estimates based on the original data, and the vertical gray dashed lines show the 95% percentile confidence limits of the bootstrap distribution. Similarly, we compared the z-values (i.e., fixed effect divided by its standard error) that are used in statistical significance assessment in Table 1 (vertical red lines) to their bootstrap distributions (histograms in bottom row; vertical dashed lines for 95% percentile CIs). The left column shows the comparisons for the centered mass effect, the middle column for the urban environment effect, and the right column for the interaction between centered mass and environment.

This analysis shows that an increase in sample size would likely result in a stronger body mass effect on larval heat knock-down time, but only in the rural environment as the interaction between centered mass and environment would also become stronger, and in a direction where the body mass effect is weaker (close to zero) in the urban environment. The main effect of ‘environment’ would likely become slightly stronger too, but the current estimate is within the 95% CIs of the bootstrap distribution, suggesting that it would be less likely that inferences concerning the ‘environment’ effect would change.


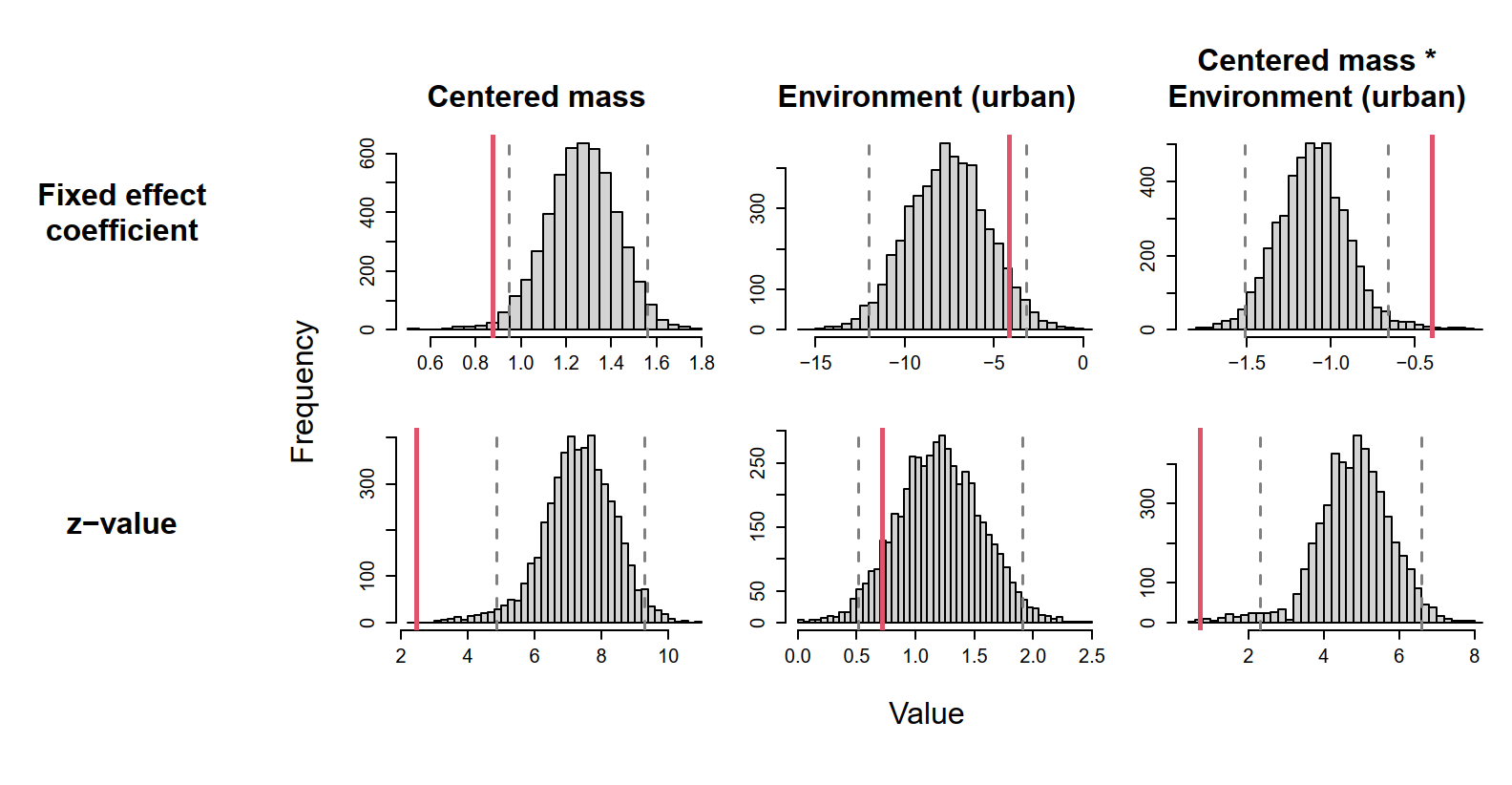


**Table S1:** Locations from which *C. clathrata* females were sampled, total numbers of full-sib families (N_FAM_) and individuals (N_TOT_) included, as well as numbers of individuals tested as larvae only (N_L_), tested as adults only (N_A_), and tested both as larvae and adults (N_L+A_). Although the total number of individuals tested overall is 688, the overall total number of heat knock-down tests amounts to 769.

| Country | City/town | Location | Environment | Latitude | Longitude | N_FAM_ | N_TOT_ | N_L_ | N_L+A_ | N_A_ |
| --- | --- | --- | --- | --- | --- | --- | --- | --- | --- | --- |
| Belgium | Brussels | Josaphat | urban | 50.863°N | 4.395°E | 7 | 118 | 9 | 16 | 93 |
| Belgium | Nismes | Regniessart | rural | 50.076°N | 4.532°E | 7 | 90 | 10 | 17 | 63 |
| Czech Republic | Prague | Pohořelec | urban | 50.087°N | 14.392°E | 3 | 48 | 7 | 5 | 36 |
| Czech Republic | Prague | Dívčí Hrady | semi-urban | 50.051°N | 14.402°E | 6 | 86 | 5 | 13 | 68 |
| Czech Republic | Milovice | Milovice NR | rural | 50.252°N | 14.887°E | 7 | 102 | 13 | 10 | 79 |
| Finland | Helsinki | Malmi | urban | 60.246°N | 25.045°E | 10 | 131 | 9 | 16 | 106 |
| Finland | Somero | Häntälä | rural | 60.576°N | 23.351°E | 10 | 113 | 29 | 4 | 80 |
| TOTAL |  |  |  |  |  | **50** | **688** | **82** | **81** | **525** |

**Table S2:** Model-averaged fixed effects for testing the effects of rearing origin of individuals on their adult HKDT. The global model included rearing ‘Origin’ and its interaction with each of age, environment, sex, and region, as well as the main effects of age, environment, sex, and region as fixed effects. 22 models were included in model averaging with the same criteria as explained for the main analyses in the main text. Two rearing origins had to be excluded from this analysis because they included populations only from one region, and inclusion of them resulted in serious estimation problems of the model parameters. The reference rearing origin were the individuals specifically reared for this study. The model output shows that none of the interactions were significant, meaning that excluding rearing origin from the main analysis did not confound results.

| **Model parameter** | **Estimate** | **Adjusted SE** | **Z value** | **P value** |
| --- | --- | --- | --- | --- |
| Intercept | 108 | 5.26 | 20.6 | <0.0001 |
| Environment (urban) | 2.10 | 2.84 | 0.738 | 0.46 |
| Region (North-European) | -7.51 | 5.45 | 1.38 | 0.17 |
| Sex (male) | -36.1 | 5.05 | 7.14 | <0.0001 |
| Origin (ALAN) | -23.3 | 6.17 | 3.78 | 0.00016 |
| Origin (dark nights) | -13.3 | 8.39 | 1.58 | 0.11 |
| Age | 0.0243 | 3.41 | 0.007 | 0.99 |
| Region (North-European) × Origin (ALAN) | 11.3 | 7.99 | 1.42 | 0.16 |
| Region (North-European) × Origin (dark nights) | 11.7 | 9.69 | 1.21 | 0.23 |
| Sex (male) × Origin (ALAN) | 12.2 | 7.46 | 1.63 | 0.10 |
| Sex (male) × Origin (dark nights) | 8.26 | 7.06 | 1.17 | 0.24 |
| Environment (urban) × Origin (ALAN) | 0.0486 | 1.12 | 0.043 | 0.97 |
| Environment (urban) × Origin (dark nights) | 0.500 | 2.53 | 0.198 | 0.84 |
| Age × Origin (ALAN) | -0.133 | 3.42 | 0.039 | 0.97 |
| Age × Origin (dark nights) | -0.0769 | 3.36 | 0.023 | 0.98 |

Rearing origins: ‘ALAN’, testing effects of dim light pollution on growth and development in Mid-European (16L:8D photoperiod) and North-European (20.5L:3.5D photoperiod) populations by exposing larvae to a dim light at night (see Merckx et al., 2023 for more details); ‘dark nights’, control treatment for the above-mentioned light pollution simulation where nights were dark (see Merckx et al., 2023 for more details); other rearing origins excluded from this analysis due to problems in model fitting include an immunological experiment measuring the effects of an artificial implant, a puncture and unmanipulated control (20L:4D photoperiod; all Mid-European individuals), and overwintered F2 individuals (12L:12D photoperiod; all North-European individuals).

**Table S3:** Table of all 10 models derived from the global model (including the global model) explaining variation in heat knock-down times in larval *C. clathrata*. Each row corresponds to one model, the first five columns indicating whether the term in question was included in the model (estimate indicated for intercept and centered mass; “+” indicates the inclusion of a factor or an interaction), the remaining columns showing model comparison metrics. Models highlighted in grey were used in model averaging (see Methods).

| **Intercept** | **Centered mass** | **Environment** | **Region** | **Centered mass × environment** | **df** | **AICc** | **ΔAICc** | **Akaike weight** |
| --- | --- | --- | --- | --- | --- | --- | --- | --- |
| 132.099 | 1.142 | + |  | + | 7.000 | 1617.642 | 0.000 | 0.312 |
| 127.897 | 1.231 | + | + | + | 8.000 | 1618.103 | 0.461 | 0.248 |
| 123.915 | 0.760 |  | + |  | 6.000 | 1619.172 | 1.530 | 0.145 |
| 128.367 | 0.628 |  |  |  | 5.000 | 1619.356 | 1.714 | 0.133 |
| 131.172 | 0.632 | + |  |  | 6.000 | 1620.471 | 2.829 | 0.076 |
| 126.583 | 0.757 | + | + |  | 7.000 | 1620.528 | 2.886 | 0.074 |
| 127.490 |  |  |  |  | 4.000 | 1625.574 | 7.932 | 0.006 |
| 130.040 |  | + |  |  | 5.000 | 1626.846 | 9.205 | 0.003 |
| 126.664 |  |  | + |  | 5.000 | 1627.616 | 9.974 | 0.002 |
| 129.381 |  | + | + |  | 6.000 | 1628.956 | 11.314 | 0.001 |

**Table S4:** Same as Table 1, except excluding the semi-urban Dívčí Hrady population from the analysis. Model-averaged fixed effects (full average) of generalized linear mixed-effects models, explaining heat knock-down time in larval *C. clathrata* in relation to environment of origin (rural/urban) and centered body mass at time of testing (centered mass).

| **Model parameter** | **Estimate** | **Adjusted SE** | **Z value** | **P value** |
| --- | --- | --- | --- | --- |
| Intercept | 130 | 4.36 | 29.7 | <.0001 |
| Environment (urban) | -1.94 | 4.86 | 0.399 | 0.69 |
| Centered mass | 0.853 | 0.342 | 2.50 | 0.013 |
| Region (North-European) | 1.64 | 4.30 | 0.380 | 0.70 |
| Environment (urban) × Centered mass | -0.331 | 0.522 | 0.635 | 0.53 |

**Table S5.** Table of all 76 models derived from the global model (including the global model) explaining variation in heat knock-down times in adult *C. clathrata*. Each row corresponds to one model, the first 10 columns indicating whether the term in question was included in the model (estimate indicated for intercept and age; “+” indicates the inclusion of a factor or an interaction), the remaining columns showing model comparison metrics. Models highlighted in grey were used in model averaging (see Methods).

| **Intercept** | **Age** | **Environment** | **Region** | **Sex** | **Tested as a larva** | **Age × environment** | **Age × sex** | **Environment × sex** | **Age × environment × sex** | **df** | **AICc** | **ΔAICc** | **Akaike weight** |
| --- | --- | --- | --- | --- | --- | --- | --- | --- | --- | --- | --- | --- | --- |
| 102.79 | -6.63 | + | + | + |  |  | + |  |  | 11.00 | 5614.91 | 0.00 | 0.36 |
| 102.33 | -6.40 | + | + | + |  | + | + |  |  | 12.00 | 5616.80 | 1.89 | 0.14 |
| 102.54 | -6.59 | + | + | + | + |  | + |  |  | 12.00 | 5616.85 | 1.94 | 0.14 |
| 102.80 | -6.63 | + | + | + |  |  | + | + |  | 12.00 | 5616.99 | 2.08 | 0.13 |
| 102.07 | -6.36 | + | + | + | + | + | + |  |  | 13.00 | 5618.73 | 3.82 | 0.05 |
| 102.40 | -6.41 | + | + | + |  | + | + | + |  | 13.00 | 5618.89 | 3.97 | 0.05 |
| 102.62 | -6.60 | + | + | + | + |  | + | + |  | 13.00 | 5618.93 | 4.02 | 0.05 |
| 101.04 | -5.60 | + | + | + |  | + | + | + | + | 14.00 | 5619.61 | 4.70 | 0.03 |
| 102.19 | -6.37 | + | + | + | + | + | + | + |  | 14.00 | 5620.82 | 5.91 | 0.02 |
| 100.87 | -5.58 | + | + | + | + | + | + | + | + | 15.00 | 5621.58 | 6.67 | 0.01 |
| 106.88 | -6.76 |  | + | + |  |  | + |  |  | 10.00 | 5622.26 | 7.35 | 0.01 |
| 99.26 | -6.68 | + |  | + |  |  | + |  |  | 10.00 | 5624.00 | 9.09 | 0.00 |
| 106.60 | -6.72 |  | + | + | + |  | + |  |  | 11.00 | 5624.16 | 9.24 | 0.00 |
| 98.91 | -6.62 | + |  | + | + |  | + |  |  | 11.00 | 5625.65 | 10.74 | 0.00 |
| 98.73 | -6.40 | + |  | + |  | + | + |  |  | 11.00 | 5625.78 | 10.87 | 0.00 |
| 99.09 | -6.67 | + |  | + |  |  | + | + |  | 11.00 | 5626.06 | 11.15 | 0.00 |
| 98.35 | -6.33 | + |  | + | + | + | + |  |  | 12.00 | 5627.42 | 12.51 | 0.00 |
| 98.84 | -6.62 | + |  | + | + |  | + | + |  | 12.00 | 5627.73 | 12.82 | 0.00 |
| 98.62 | -6.40 | + |  | + |  | + | + | + |  | 12.00 | 5627.86 | 12.95 | 0.00 |
| 97.23 | -5.55 | + |  | + |  | + | + | + | + | 13.00 | 5628.41 | 13.50 | 0.00 |
| 98.35 | -6.33 | + |  | + | + | + | + | + |  | 13.00 | 5629.51 | 14.60 | 0.00 |
| 103.37 | -6.79 |  |  | + |  |  | + |  |  | 9.00 | 5629.74 | 14.83 | 0.00 |
| 96.99 | -5.51 | + |  | + | + | + | + | + | + | 14.00 | 5630.12 | 15.21 | 0.00 |
| 103.00 | -6.73 |  |  | + | + |  | + |  |  | 10.00 | 5631.39 | 16.48 | 0.00 |
| 97.75 | -2.91 | + | + | + |  |  |  |  |  | 10.00 | 5633.60 | 18.68 | 0.00 |
| 96.90 | -2.90 | + | + | + |  |  |  | + |  | 11.00 | 5635.33 | 20.41 | 0.00 |
| 97.52 | -2.88 | + | + | + | + |  |  |  |  | 11.00 | 5635.52 | 20.61 | 0.00 |
| 97.43 | -2.74 | + | + | + |  | + |  |  |  | 11.00 | 5635.57 | 20.66 | 0.00 |
| 96.75 | -2.88 | + | + | + | + |  |  | + |  | 12.00 | 5637.30 | 22.39 | 0.00 |
| 96.65 | -2.76 | + | + | + |  | + |  | + |  | 12.00 | 5637.34 | 22.42 | 0.00 |
| 97.19 | -2.72 | + | + | + | + | + |  |  |  | 12.00 | 5637.50 | 22.59 | 0.00 |
| 96.49 | -2.74 | + | + | + | + | + |  | + |  | 13.00 | 5639.31 | 24.40 | 0.00 |
| 101.88 | -3.02 |  | + | + |  |  |  |  |  | 9.00 | 5641.14 | 26.23 | 0.00 |
| 101.62 | -2.99 |  | + | + | + |  |  |  |  | 10.00 | 5643.04 | 28.13 | 0.00 |
| 93.80 | -2.81 | + |  | + |  |  |  |  |  | 9.00 | 5644.82 | 29.91 | 0.00 |
| 92.76 | -2.81 | + |  | + |  |  |  | + |  | 10.00 | 5646.29 | 31.38 | 0.00 |
| 93.46 | -2.77 | + |  | + | + |  |  |  |  | 10.00 | 5646.42 | 31.51 | 0.00 |
| 93.41 | -2.60 | + |  | + |  | + |  |  |  | 10.00 | 5646.73 | 31.82 | 0.00 |
| 92.54 | -2.78 | + |  | + | + |  |  | + |  | 11.00 | 5648.00 | 33.09 | 0.00 |
| 92.45 | -2.63 | + |  | + |  | + |  | + |  | 11.00 | 5648.25 | 33.34 | 0.00 |
| 93.04 | -2.55 | + |  | + | + | + |  |  |  | 11.00 | 5648.33 | 33.41 | 0.00 |
| 92.20 | -2.58 | + |  | + | + | + |  | + |  | 12.00 | 5649.95 | 35.04 | 0.00 |
| 97.96 | -2.92 |  |  | + |  |  |  |  |  | 8.00 | 5650.36 | 35.44 | 0.00 |
| 97.61 | -2.88 |  |  | + | + |  |  |  |  | 9.00 | 5651.96 | 37.05 | 0.00 |
| 92.95 |  | + | + | + |  |  |  |  |  | 9.00 | 5652.11 | 37.20 | 0.00 |
| 92.52 |  | + | + | + | + |  |  |  |  | 10.00 | 5653.50 | 38.59 | 0.00 |
| 92.03 |  | + | + | + |  |  |  | + |  | 10.00 | 5653.81 | 38.90 | 0.00 |
| 91.74 |  | + | + | + | + |  |  | + |  | 11.00 | 5655.30 | 40.38 | 0.00 |
| 97.53 |  |  | + | + |  |  |  |  |  | 8.00 | 5660.71 | 45.79 | 0.00 |
| 89.11 |  | + |  | + |  |  |  |  |  | 8.00 | 5661.29 | 46.38 | 0.00 |
| 97.06 |  |  | + | + | + |  |  |  |  | 9.00 | 5662.02 | 47.11 | 0.00 |
| 88.66 |  | + |  | + | + |  |  |  |  | 9.00 | 5662.21 | 47.30 | 0.00 |
| 88.03 |  | + |  | + |  |  |  | + |  | 9.00 | 5662.79 | 47.88 | 0.00 |
| 87.75 |  | + |  | + | + |  |  | + |  | 10.00 | 5663.86 | 48.95 | 0.00 |
| 93.73 |  |  |  | + |  |  |  |  |  | 7.00 | 5668.00 | 53.09 | 0.00 |
| 93.24 |  |  |  | + | + |  |  |  |  | 8.00 | 5668.91 | 54.00 | 0.00 |
| 84.40 | -3.23 | + | + |  |  |  |  |  |  | 9.00 | 5762.96 | 148.05 | 0.00 |
| 84.16 | -3.20 | + | + |  | + |  |  |  |  | 10.00 | 5764.78 | 149.87 | 0.00 |
| 84.04 | -3.04 | + | + |  |  | + |  |  |  | 10.00 | 5764.92 | 150.01 | 0.00 |
| 83.78 | -3.00 | + | + |  | + | + |  |  |  | 11.00 | 5766.73 | 151.82 | 0.00 |
| 88.52 | -3.39 |  | + |  |  |  |  |  |  | 8.00 | 5769.37 | 154.46 | 0.00 |
| 88.27 | -3.37 |  | + |  | + |  |  |  |  | 9.00 | 5771.16 | 156.25 | 0.00 |
| 81.10 | -3.22 | + |  |  |  |  |  |  |  | 8.00 | 5776.49 | 161.58 | 0.00 |
| 80.75 | -3.18 | + |  |  | + |  |  |  |  | 9.00 | 5778.06 | 163.15 | 0.00 |
| 80.51 | -2.88 | + |  |  |  | + |  |  |  | 9.00 | 5778.20 | 163.29 | 0.00 |
| 80.14 | -2.83 | + |  |  | + | + |  |  |  | 10.00 | 5779.75 | 164.84 | 0.00 |
| 85.01 | -3.36 |  |  |  |  |  |  |  |  | 7.00 | 5780.27 | 165.36 | 0.00 |
| 84.67 | -3.32 |  |  |  | + |  |  |  |  | 8.00 | 5781.82 | 166.91 | 0.00 |
| 77.79 |  | + | + |  |  |  |  |  |  | 8.00 | 5783.08 | 168.17 | 0.00 |
| 77.42 |  | + | + |  | + |  |  |  |  | 9.00 | 5784.23 | 169.32 | 0.00 |
| 82.39 |  |  | + |  |  |  |  |  |  | 7.00 | 5790.96 | 176.05 | 0.00 |
| 82.00 |  |  | + |  | + |  |  |  |  | 8.00 | 5792.04 | 177.13 | 0.00 |
| 73.94 |  | + |  |  |  |  |  |  |  | 7.00 | 5794.99 | 180.08 | 0.00 |
| 73.50 |  | + |  |  | + |  |  |  |  | 8.00 | 5795.75 | 180.84 | 0.00 |
| 78.43 |  |  |  |  |  |  |  |  |  | 6.00 | 5800.27 | 185.35 | 0.00 |
| 77.99 |  |  |  |  | + |  |  |  |  | 7.00 | 5800.98 | 186.07 | 0.00 |

**Table S6:** Same as Table 2, except excluding the semi-urban Dívčí Hrady population from the analysis. Model-averaged fixed effects (full average) of linear mixed effects models explaining variation in heat knock-down time in adult *C. clathrata* in relation to age since adult eclosion (days), environment of origin (rural/urban), region (North-European/Mid-European), sex, and an indicator variable for whether the same individual had also been measured as a larva.

| **Model parameter** | **Estimate** | **Adjusted SE** | **Z value** | **P value** |
| --- | --- | --- | --- | --- |
| Intercept | 104 | 2.96 | 35.1 | <0.0001 |
| Age | -6.72 | 1.11 | 6.03 | <0.0001 |
| Environment (urban) | 7.21 | 2.75 | 2.62 | 0.0087 |
| Region (North-European) | -8.23 | 2.46 | 3.34 | 0.00083 |
| Sex (male) | -34.6 | 2.90 | 11.9 | <0.0001 |
| Age × Sex (male) | 5.69 | 1.36 | 4.19 | <0.0001 |

**R scripts of analyses**

## Larvae

## ------

library(nlme)

library(MuMIn)

# centered mass

larvae$Cmass<-larvae$mass - mean(larvae$mass)

l0<-lme(HKDT~Cmass*environment + region,

random=~1|family,

data=larvae,

method="ML")

AICc(l0)

summary(l0)

intervals(l0) # family-level variance estimates inaccurately

plot(l0)

qqnorm(l0, ~resid(.))

qqnorm(l0, ~ranef(.))

plot(l0, sex~resid(.))

plot(l0, Population~resid(.))

plot(l0, region~resid(.)) # more residual variance in the Mid-European populations

plot(l0, environment~resid(.))

hist(resid(l0))

# add a variance function to model heteroscedasticity

l0b<-lme(HKDT~Cmass*environment + region,

random=~1|family,

data=larvae,

method="ML",

weights=varIdent(form=~1|region))

AICc(l0b) - AICc(l0) # l0b is 8.3 AICc units better

anova(l0, l0b)

summary(l0b)

intervals(l0b) # family-level variance estimates inaccurately, but better than in model l0

plot(l0b)

qqnorm(l0b, ~resid(.))

qqnorm(l0b, ~ranef(.))

plot(l0b, sex~resid(.))

plot(l0b, Population~resid(.))

plot(l0b, region~resid(.))

plot(l0b, environment~resid(.))

plot(l0b, resid(.)~Mass)

hist(resid(l0b))

# Try an exponential variance function instead

l0c<-lme(HKDT~Cmass*environment + region,

random=~1|family,

data=larvae,

method="ML",

weights=varExp(form=~fitted(.)))

AICc(l0c) - AICc(l0b) # l0c is 6.1 AICc units worse

anova(l0b, l0c)

#####

# Model averaging (global model = l0b)

models.l0b<-dredge(global.model=l0b, beta="none", rank="AICc")

model.table.l0b<-model.sel(models.l0b)

write.csv2(model.table.l0b, file="model.table.l0b.csv")

model.table.l0b

# There are 7 models (out of 10) that are within 8 AICc units from the best model.

# One of these seven models is more complex (i.e. include more parameters) than the best model.

# The best model is not superior to the other models (AICc difference between two best models is 0.46),

# so model averaging seems reasonable, and a meaningful model set for averaging includes those models

# that are <8 AICc units from the best model and are not more complex than the best model. This

# leaves six models for averaging.

models.l0b.avg<-get.models(models.l0b, subset = delta < 8 & df<=7)

avg.model.l0b<-model.avg(models.l0b.avg, fit=TRUE)

summary(avg.model.l0b)

###

# Bootstrap CIs for predictions

# .............................

# Omit the 'region' variable here, for simplicity, because it did not

# explain anything in the analysis above, and no region-level variation

# will be visualized in the figure.

# Number of bootstrap resamples

B<-5000

massseq<-seq(min(larvae$Cmass), max(larvae$Cmass), len=100)

Boot.U<-matrix(nrow=length(massseq), ncol=B)

Boot.R<-matrix(nrow=length(massseq), ncol=B)

uniqs<-unique(larvae[,c("environment")])

larvae$Uniqs<-numeric(nrow(larvae))

numseq<-1:length(uniqs)

for(n in 1:length(uniqs)){

larvae$Uniqs[{larvae$environment==uniqs[n]}] <- numseq[n]

}

set.seed(12)

larvae$Boot.response<-numeric(nrow(larvae))

larvae$Boot.Cmass<-numeric(nrow(larvae))

for(i in 1:B){

for(k in 1:length(uniqs)){

numseq2<-1:length(larvae$Uniqs[larvae$Uniqs==numseq[k]])

index<-sample(numseq2, size=length(larvae$HKDT[larvae$Uniqs==numseq[k]]),

replace=TRUE)

larvae$Boot.response[larvae$Uniqs==numseq[k]] <- larvae$HKDT[larvae$Uniqs==numseq[k]][index]

larvae$Boot.Cmass[larvae$Uniqs==numseq[k]] <- larvae$Cmass[larvae$Uniqs==numseq[k]][index]

}

model<-lme(Boot.response~Boot.Cmass*environment,

random=~1|family,

data=larvae,

method="ML",

weights=varIdent(form=~1|region))

# Note that all models derived from the 'model' are included in model averaging here.

# This is because the model averaging otherwise fails, as there will be such bootstrap

# realizations of the data where only a single model is left with the criteria of

# delta < 8 & df<=7. Consequently, the CIs are conservative to some degree.

models<-dredge(global.model=model, beta="none", rank="AICc")

avg.model<-model.avg(models, fit=TRUE)

# Rural

Rp<-avg.model$coefficients[1,"(Intercept)"] +

avg.model$coefficients[1,"Boot.Cmass"]*massseq

# Urban

Up<-avg.model$coefficients[1,"(Intercept)"] + avg.model$coefficients[1,"environmenturban"] +

(avg.model$coefficients[1,"Boot.Cmass"] + avg.model$coefficients[1,"Boot.Cmass:environmenturban"])*massseq

Boot.R[,i]<-Rp

Boot.U[,i]<-Up

}

# Derive percentile CIs

R.CIL<-numeric(length(massseq))

R.CIU<-numeric(length(massseq))

U.CIL<-numeric(length(massseq))

U.CIU<-numeric(length(massseq))

for(j in 1:length(massseq)){

quant.R<-quantile(Boot.R[j,], probs=c(0.025, 0.975))

R.CIL[j]<-quant.R[1]

R.CIU[j]<-quant.R[2]

quant.U<-quantile(Boot.U[j,], probs=c(0.025, 0.975))

U.CIL[j]<-quant.U[1]

U.CIU[j]<-quant.U[2]

}

# Use means of bootstrap distributions as point estimates

R.fit<-rowMeans(Boot.R)

U.fit<-rowMeans(Boot.U)

#####

# Figure

# ......

rural.col<-"#E69F00"

urban.col<-"#0072B2"

Rur<-subset(larvae, environment=="rural")

Urb<-subset(larvae, environment=="urban")

massseq.orig<-seq(min(larvae$mass), max(larvae$mass), len=100)

par(mfrow=c(1,1), mar=c(5,5,2,2))

plot(Rur$HKDT~Rur$mass, type="p", pch=as.numeric(Rur$region), lwd=2, cex=2, col=rural.col,

xlim=range(larvae$mass),

ylim=range(larvae$HKDT),

xlab="Mass (mg)",

ylab="Heat knock-down time (s)",

cex.lab=1.5, cex.axis=1.3)

points(Urb$HKDT~Urb$mass, type="p", pch=as.numeric(Urb$region), lwd=2, cex=2, col=urban.col)

lines(R.fit~massseq.orig, lwd=3, lty=1, col=rural.col)

lines(U.fit~massseq.orig, lwd=3, lty=1, col=urban.col)

xcoords<-massseq.orig

polygon(x=c(xcoords, rev(xcoords)),

y=c(R.CIL, rev(R.CIU)),

col=adjustcolor(rural.col, alpha.f = 0.30),

border = NA)

polygon(x=c(xcoords, rev(xcoords)),

y=c(U.CIL, rev(U.CIU)),

col=adjustcolor(urban.col, alpha.f = 0.30),

border = NA)

legend("topleft", legend=c("rural", "urban", "Mid-European", "North-European"),

lty=c(1, 1, NA, NA), lwd=c(3, 3, NA, NA), col=c(rural.col, urban.col, 1, 1),

pch=c(NA, NA, 1, 2), cex=1.3, pt.cex=2)

#####

# The analysis without the Divci Hrady population was repeated with the above procedure

# but just replacing the data with a subset where the Divci Hrady population is excluded.

####################################################################################################

## Adults

## ------

# Test the effect of rearing origin (source) of tested individuals on HKDT

# ................................................................

# Set HKDT rearing as the reference category

adults$Source<-factor(adults$Source)

adults$Source<-relevel(adults$Source, ref="HKDT")

library(lattice)

bwplot(HKDT~region|Source, data=adults)

bwplot(HKDT~sex|Source, data=adults)

bwplot(HKDT~environment|Source, data=adults)

# Visual assessment does not suggest interactions including Source

# Region*Source interaction cannot be included in the model, because

# some sources are region-specific.

# All data (without region)

s0<-lme(HKDT~environment*Source + sex*Source + Age*Source,

random=~1|family,

data=adults,

method="ML")

summary(s0)

intervals(s0) # cannot derive CIs

plot(s0)

qqnorm(s0, ~resid(.))

qqnorm(s0, ~ranef(.))

s0b<-lme(HKDT~environment*Source + sex*Source + Age*Source,

random=~1|family,

data=adults,

method="ML",

weights=varIdent(form=~1|region))

AICc(s0b) - AICc(s0)

summary(s0b)

intervals(s0b) # cannot derive CIs

plot(s0b)

qqnorm(s0b, ~resid(.))

qqnorm(s0b, ~ranef(.))

###

# Immuno and Stock sources ignored, because no region-level replication in

# these sources

adults.2<-adults[adults$Source!="Stock",]

adults.3<-adults.2[adults.2$Source!="Immuno",]

s1<-lme(HKDT~environment*Source + sex*Source + region*Source + Age*Source,

random=~1|family,

data=adults.3,

method="ML")

summary(s1)

intervals(s1) # family-level variance inaccurate

plot(s1)

qqnorm(s1, ~resid(.))

qqnorm(s1, ~ranef(.))

plot(s1, sex~resid(.))

plot(s1, Population~resid(.))

plot(s1, region~resid(.))

plot(s1, environment~resid(.))

s2<-lme(HKDT~environment*Source + sex*Source + region*Source + Age*Source,

random=~1|family,

data=adults.3,

method="ML",

weights=varIdent(form=~1|region*sex))

AICc(s2) - AICc(s1) # Model s2 is 48.1 AICc units better

anova(s1, s2)

summary(s2)

intervals(s2) # family-level variance still a bit inaccurate, but much better than in s1

plot(s2)

qqnorm(s2, ~resid(.))

qqnorm(s2, ~ranef(.))

plot(s2, sex~resid(.))

plot(s2, Population~resid(.))

plot(s2, region~resid(.))

plot(s2, environment~resid(.))

models.s2<-dredge(global.model=s2, beta="none", rank="AICc")

model.table.s2<-model.sel(models.s2)

model.table.s2

models.s2.avg<-get.models(models.s2, subset = delta < 8 & df<=15)

avg.model.s2<-model.avg(models.s2.avg, fit=TRUE)

summary(avg.model.s2)

# Analysis of HKDT in relation to the factors of interest

# .......................................................

library(nlme)

library(MuMIn)

a0<-lme(HKDT~Age*environment*sex + environment*region + Tested.as.larva,

random=~1|family,

data=adults,

method="ML")

AICc(a0)

summary(a0)

intervals(a0) # family-level variance extremely inaccurate; estimation problems

plot(a0)

qqnorm(a0, ~resid(.))

qqnorm(a0, ~ranef(.))

plot(a0, sex~resid(.))

plot(a0, Population~resid(.))

plot(a0, region~resid(.))

plot(a0, environment~resid(.))

plot(resid(a0)~adults$Age)

# Heteroscedasticity in a0, so add a variance function to take it into account

a0b<-lme(HKDT~Age*environment*sex + environment*region + Tested.as.larva,

random=~1|family,

data=adults,

method="ML",

weights=varIdent(form=~1|region*sex))

AICc(a0b) - AICc(a0) # a0b is 33.9 AICc units better

anova(a0, a0b)

summary(a0b)

intervals(a0b) # Estimate for family-level variance uncertain

plot(a0b)

qqnorm(a0b, ~resid(.))

qqnorm(a0b, ~ranef(.))

plot(a0b, sex~resid(.))

plot(a0b, Population~resid(.))

plot(a0b, region~resid(.))

plot(a0b, environment~resid(.))

plot(resid(a0b)~adults$Age)

hist(resid(a0b))

# Try a different variance function

a0c<-lme(HKDT~Age*environment*sex + environment*region + Tested.as.larva,

random=~1|family,

data=adults,

method="ML",

weights=varExp(form=~Age))

AICc(a0c) - AICc(a0b) # a0c is 35.0 AICc units worse

anova(a0b, a0c)

summary(a0c)

intervals(a0c) # Estimate for family-level variance extremely uncertain

plot(a0c)

qqnorm(a0c, ~resid(.))

qqnorm(a0c, ~ranef(.))

plot(a0c, sex~resid(.))

plot(a0c, Population~resid(.))

plot(a0c, region~resid(.))

plot(a0c, environment~resid(.))

plot(resid(a0c)~adults$Age)

hist(resid(a0c))

# As there is clear heteroscedasticity in relation to age, try combining modelling

# residual variance both in relation to age and the combimations of region and sex

a0d<-lme(HKDT~Age*environment*sex + environment*region + Tested.as.larva,

random=~1|family,

data=adults,

method="ML",

weights=varComb(varExp(form=~Age), varIdent(form=~1|region*sex)))

AICc(a0d) - AICc(a0b) # a0d is 1.88 AICc units better

anova(a0b, a0d)

summary(a0d)

intervals(a0d) # Estimate for family-level variance uncertain

plot(a0d)

qqnorm(a0d, ~resid(.))

qqnorm(a0d, ~ranef(.))

plot(a0d, sex~resid(.))

plot(a0d, Population~resid(.))

plot(a0d, region~resid(.))

plot(a0d, environment~resid(.))

plot(resid(a0d)~adults$Age)

hist(resid(a0d))

# Because family-level variance estimates inaccurately and there is no evidence of

# an interaction between environment and region, let's try simplifying the model

# a bit by removing the environment*region, but retaining the main effect of region,

# to see if it improves estimation accuracy of model parameters.

a1<-lme(HKDT~Age*environment*sex + region + Tested.as.larva,

random=~1|family,

data=adults,

method="ML",

weights=varIdent(form=~1|region*sex))

AICc(a1) - AICc(a0b) # a1 is 1.22 AICc units worse

anova(a0b, a1)

summary(a1)

intervals(a1) # Estimate for family-level variance still uncertain, but better than in model a0b

plot(a1)

qqnorm(a1, ~resid(.))

qqnorm(a1, ~ranef(.))

plot(a1, sex~resid(.))

plot(a1, Population~resid(.))

plot(a1, region~resid(.))

plot(a1, environment~resid(.))

plot(resid(a1)~adults$Age)

hist(resid(a1))

a1b<-lme(HKDT~Age*environment*sex + region + Tested.as.larva,

random=~1|family,

data=adults,

method="ML",

weights=varComb(varExp(form=~Age), varIdent(form=~1|region*sex)))

AICc(a1b) - AICc(a1) # a1b is 1.91 AICc units better

anova(a1, a1b)

summary(a1b)

intervals(a1b) # Estimate for family-level variance still uncertain, but better than in model a0b

plot(a1b)

qqnorm(a1b, ~resid(.))

qqnorm(a1b, ~ranef(.))

plot(a1b, sex~resid(.))

plot(a1b, Population~resid(.))

plot(a1b, region~resid(.))

plot(a1b, environment~resid(.))

plot(resid(a1b)~adults$Age)

hist(resid(a1b))

# The difference between models a0b and a1 in AICc value is so small that they are

# practically equally good. As the family-level variance estimates a bit better in

# model a1 than in a0b - and diagnostics are otherwise essentially identical - it

# seems reasonable to use the model a1 as a global model in model averaging.

#####

# Model averaging (global model = a1)

models.a1<-dredge(global.model=a1, beta="none", rank="AICc")

model.table.a1<-model.sel(models.a1)

write.csv2(model.table.a1, file="model.table.a1.csv")

model.table.a1

# There are 11 models (out of 76) that are within 8 AICc units from the best model.

# Nine of these 11 models are more complex (i.e. include more parameters) than the best model.

# The best model is not superior to the other models in this comparison (AICc difference between two

# best models is 1.89), so model averaging seems reasonable, and a meaningful model set for averaging

# includes those models that are <8 AICc units from the best model and are not more complex than the best model. This

# leaves two models for averaging.

models.a1.avg<-get.models(models.a1, subset = delta < 8 & df<=11)

avg.model.a1<-model.avg(models.a1.avg, fit=TRUE)

summary(avg.model.a1)

###

# Bootstrap CIs for predictions

# .............................

# Number of bootstrap resamples

B<-5000

ageseq<-seq(min(adults$Age), max(adults$Age), len=100)

Boot.RFmid<-matrix(nrow=length(ageseq), ncol=B)

Boot.RMmid<-matrix(nrow=length(ageseq), ncol=B)

Boot.UFmid<-matrix(nrow=length(ageseq), ncol=B)

Boot.UMmid<-matrix(nrow=length(ageseq), ncol=B)

Boot.RFnor<-matrix(nrow=length(ageseq), ncol=B)

Boot.RMnor<-matrix(nrow=length(ageseq), ncol=B)

Boot.UFnor<-matrix(nrow=length(ageseq), ncol=B)

Boot.UMnor<-matrix(nrow=length(ageseq), ncol=B)

uniqs<-unique(adults[,c("environment", "sex", "region")])

adults$Uniqs<-numeric(nrow(adults))

numseq<-1:nrow(uniqs)

for(n in 1:nrow(uniqs)){

adults$Uniqs[{adults$environment==uniqs$environment[n] &

adults$sex==uniqs$sex[n] &

adults$region==uniqs$region[n] }] <- numseq[n]

}

set.seed(12)

adults$Boot.response<-numeric(nrow(adults))

for(i in 1:B){

for(k in 1:nrow(uniqs)){

numseq2<-1:length(adults$Uniqs[adults$Uniqs==numseq[k]])

index<-sample(numseq2, size=length(adults$HKDT[adults$Uniqs==numseq[k]]),

replace=TRUE)

adults$Boot.response[adults$Uniqs==numseq[k]] <- adults$HKDT[adults$Uniqs==numseq[k]][index]

adults$Boot.age[adults$Uniqs==numseq[k]] <- adults$Age[adults$Uniqs==numseq[k]][index]

}

m1<-lme(Boot.response~Boot.age + environment + region + sex + Boot.age:sex,

random=~1|family,

data=adults,

method="ML",

weights=varIdent(form=~1|region*sex))

m2<-lme(Boot.response~Boot.age + region + sex + Boot.age:sex,

random=~1|family,

data=adults,

method="ML",

weights=varIdent(form=~1|region*sex))

avg.model<-model.avg(list(m1, m2), fit=TRUE)

# Mid-European

RFm<-avg.model$coefficients[1,"(Intercept)"] +

avg.model$coefficients[1,"Boot.age"]*ageseq

RMm<-avg.model$coefficients[1,"(Intercept)"] + avg.model$coefficients[1,"sexmale"] +

(avg.model$coefficients[1,"Boot.age"] + avg.model$coefficients[1,"Boot.age:sexmale"])*ageseq

UFm<-avg.model$coefficients[1,"(Intercept)"] + avg.model$coefficients[1,"environmenturban"] +

avg.model$coefficients[1,"Boot.age"]*ageseq

UMm<-avg.model$coefficients[1,"(Intercept)"] + avg.model$coefficients[1,"sexmale"] +

avg.model$coefficients[1,"environmenturban"] +

(avg.model$coefficients[1,"Boot.age"] + avg.model$coefficients[1,"Boot.age:sexmale"])*ageseq

# Nordic

RFn<-avg.model$coefficients[1,"(Intercept)"] + avg.model$coefficients[1,"regionNordic"] +

avg.model$coefficients[1,"Boot.age"]*ageseq

RMn<-avg.model$coefficients[1,"(Intercept)"] + avg.model$coefficients[1,"sexmale"] + avg.model$coefficients[1,"regionNordic"] +

(avg.model$coefficients[1,"Boot.age"] + avg.model$coefficients[1,"Boot.age:sexmale"])*ageseq

UFn<-avg.model$coefficients[1,"(Intercept)"] + avg.model$coefficients[1,"environmenturban"] + avg.model$coefficients[1,"regionNordic"] +

avg.model$coefficients[1,"Boot.age"]*ageseq

UMn<-avg.model$coefficients[1,"(Intercept)"] + avg.model$coefficients[1,"sexmale"] + avg.model$coefficients[1,"regionNordic"] +

avg.model$coefficients[1,"environmenturban"] +

(avg.model$coefficients[1,"Boot.age"] + avg.model$coefficients[1,"Boot.age:sexmale"])*ageseq

Boot.RFmid[,i]<-RFm

Boot.RMmid[,i]<-RMm

Boot.UFmid[,i]<-UFm

Boot.UMmid[,i]<-UMm

Boot.RFnor[,i]<-RFn

Boot.RMnor[,i]<-RMn

Boot.UFnor[,i]<-UFn

Boot.UMnor[,i]<-UMn

}

# Derive percentile CIs

RF.CILmid<-numeric(length(ageseq))

RF.CIUmid<-numeric(length(ageseq))

RM.CILmid<-numeric(length(ageseq))

RM.CIUmid<-numeric(length(ageseq))

UF.CILmid<-numeric(length(ageseq))

UF.CIUmid<-numeric(length(ageseq))

UM.CILmid<-numeric(length(ageseq))

UM.CIUmid<-numeric(length(ageseq))

RF.CILnor<-numeric(length(ageseq))

RF.CIUnor<-numeric(length(ageseq))

RM.CILnor<-numeric(length(ageseq))

RM.CIUnor<-numeric(length(ageseq))

UF.CILnor<-numeric(length(ageseq))

UF.CIUnor<-numeric(length(ageseq))

UM.CILnor<-numeric(length(ageseq))

UM.CIUnor<-numeric(length(ageseq))

###

for(j in 1:length(ageseq)){

quant.RFmid<-quantile(Boot.RFmid[j,], probs=c(0.025, 0.975))

RF.CILmid[j]<-quant.RFmid[1]

RF.CIUmid[j]<-quant.RFmid[2]

quant.RMmid<-quantile(Boot.RMmid[j,], probs=c(0.025, 0.975))

RM.CILmid[j]<-quant.RMmid[1]

RM.CIUmid[j]<-quant.RMmid[2]

quant.UFmid<-quantile(Boot.UFmid[j,], probs=c(0.025, 0.975))

UF.CILmid[j]<-quant.UFmid[1]

UF.CIUmid[j]<-quant.UFmid[2]

quant.UMmid<-quantile(Boot.UMmid[j,], probs=c(0.025, 0.975))

UM.CILmid[j]<-quant.UMmid[1]

UM.CIUmid[j]<-quant.UMmid[2]

quant.RFnor<-quantile(Boot.RFnor[j,], probs=c(0.025, 0.975))

RF.CILnor[j]<-quant.RFnor[1]

RF.CIUnor[j]<-quant.RFnor[2]

quant.RMnor<-quantile(Boot.RMnor[j,], probs=c(0.025, 0.975))

RM.CILnor[j]<-quant.RMnor[1]

RM.CIUnor[j]<-quant.RMnor[2]

quant.UFnor<-quantile(Boot.UFnor[j,], probs=c(0.025, 0.975))

UF.CILnor[j]<-quant.UFnor[1]

UF.CIUnor[j]<-quant.UFnor[2]

quant.UMnor<-quantile(Boot.UMnor[j,], probs=c(0.025, 0.975))

UM.CILnor[j]<-quant.UMnor[1]

UM.CIUnor[j]<-quant.UMnor[2]

}

# Use means of bootstrap distributions as point estimates

RFmid.fit<-rowMeans(Boot.RFmid)

RMmid.fit<-rowMeans(Boot.RMmid)

UFmid.fit<-rowMeans(Boot.UFmid)

UMmid.fit<-rowMeans(Boot.UMmid)

RFnor.fit<-rowMeans(Boot.RFnor)

RMnor.fit<-rowMeans(Boot.RMnor)

UFnor.fit<-rowMeans(Boot.UFnor)

UMnor.fit<-rowMeans(Boot.UMnor)

#####

# Figure

# ......

RFmid<-subset(adults, {environment=="rural" & sex=="female" & region=="Central-European"})

RMmid<-subset(adults, {environment=="rural" & sex=="male" & region=="Central-European"})

UFmid<-subset(adults, {environment=="urban" & sex=="female" & region=="Central-European"})

UMmid<-subset(adults, {environment=="urban" & sex=="male" & region=="Central-European"})

RFnor<-subset(adults, {environment=="rural" & sex=="female" & region=="Nordic"})

RMnor<-subset(adults, {environment=="rural" & sex=="male" & region=="Nordic"})

UFnor<-subset(adults, {environment=="urban" & sex=="female" & region=="Nordic"})

UMnor<-subset(adults, {environment=="urban" & sex=="male" & region=="Nordic"})

#####

rural.col<-"#E69F00"

urban.col<-"#0072B2"

mat=matrix(c(1, 2, 3, 1, 4, 5, 0, 6, 6), 3, 3, byrow=TRUE)

nf=layout(mat, respect=TRUE, widths=c(1, 5, 5),

heights=c(4, 4, 1))

layout.show(nf)

###

par(mar=c(1, 1, 1, 1))

plot(1:2, 1:2, type="n", bty="n", xaxt="n", yaxt="n")

mtext("Heat knock-down time (s)", side=2, line=-3, cex=1.5)

###

par(mar=c(3, 3, 2, 1))

plot(RFnor$HKDT~I(RFnor$Age + 0.1), type="p", pch=1, lwd=2, cex=2, col=rural.col,

xlim=range(adults$Age),

ylim=range(adults$HKDT),

xlab="",

ylab="",

cex.lab=1.5, cex.axis=1.3,

main="North-European females", cex.main=2)

points(UFnor$HKDT~I(UFnor$Age - 0.1), type="p", pch=1, lwd=2, cex=2, col=urban.col)

RFnor.index<-ageseq>=min(RFnor$Age) & ageseq<=max(RFnor$Age)

UFnor.index<-ageseq>=min(UFnor$Age) & ageseq<=max(UFnor$Age)

lines(RFnor.fit[RFnor.index]~ageseq[RFnor.index], lwd=3, lty=1, col=rural.col)

lines(UFnor.fit[UFnor.index]~ageseq[UFnor.index], lwd=3, lty=1, col=urban.col)

abline(v=ageseq[match(FALSE, UFnor.fit>RF.CIUnor)], lty=2, col=gray(0.5), lwd=2)

xcoords<-ageseq[RFnor.index]

polygon(x=c(xcoords, rev(xcoords)),

y=c(RF.CILnor[RFnor.index], rev(RF.CIUnor[RFnor.index])),

col=adjustcolor(rural.col, alpha.f = 0.30),

border = NA)

xcoords<-ageseq[UFnor.index]

polygon(x=c(xcoords, rev(xcoords)),

y=c(UF.CILnor[UFnor.index], rev(UF.CIUnor[UFnor.index])),

col=adjustcolor(urban.col, alpha.f = 0.30),

border = NA)

legend("topright", legend=c("rural", "urban"),

lty=1, lwd=3, col=c(rural.col, urban.col), cex=1.5)

###

plot(RMnor$HKDT~I(RMnor$Age + 0.1), type="p", pch=1, lwd=2, cex=2, col=rural.col,

xlim=range(adults$Age),

ylim=range(adults$HKDT),

xlab="",

ylab="",

cex.lab=1.5, cex.axis=1.3,

main="North-European males", cex.main=2)

points(UMnor$HKDT~I(UMnor$Age - 0.1), type="p", pch=1, lwd=2, cex=2, col=urban.col)

RMnor.index<-ageseq>=min(RMnor$Age) & ageseq<=max(RMnor$Age)

UMnor.index<-ageseq>=min(UMnor$Age) & ageseq<=max(UMnor$Age)

lines(RMnor.fit[RMnor.index]~ageseq[RMnor.index], lwd=3, lty=1, col=rural.col)

lines(UMnor.fit[UMnor.index]~ageseq[UMnor.index], lwd=3, lty=1, col=urban.col)

abline(v=ageseq[match(FALSE, UMnor.fit>RM.CIUnor)], lty=2, col=gray(0.5), lwd=2)

xcoords<-ageseq[RMnor.index]

polygon(x=c(xcoords, rev(xcoords)),

y=c(RM.CILnor[RMnor.index], rev(RM.CIUnor[RMnor.index])),

col=adjustcolor(rural.col, alpha.f = 0.30),

border = NA)

xcoords<-ageseq[UMnor.index]

polygon(x=c(xcoords, rev(xcoords)),

y=c(UM.CILnor[UMnor.index], rev(UM.CIUnor[UMnor.index])),

col=adjustcolor(urban.col, alpha.f = 0.30),

border = NA)

###

plot(RFmid$HKDT~I(RFmid$Age + 0.1), type="p", pch=1, lwd=2, cex=2, col=rural.col,

xlim=range(adults$Age),

ylim=range(adults$HKDT),

xlab="",

ylab="",

cex.lab=1.5, cex.axis=1.3,

main="Mid-European females", cex.main=2)

points(UFmid$HKDT~I(UFmid$Age - 0.1), type="p", pch=1, lwd=2, cex=2, col=urban.col)

RFmid.index<-ageseq>=min(RFmid$Age) & ageseq<=max(RFmid$Age)

UFmid.index<-ageseq>=min(UFmid$Age) & ageseq<=max(UFmid$Age)

lines(RFmid.fit[RFmid.index]~ageseq[RFmid.index], lwd=3, lty=1, col=rural.col)

lines(UFmid.fit[UFmid.index]~ageseq[UFmid.index], lwd=3, lty=1, col=urban.col)

abline(v=ageseq[match(FALSE, UFmid.fit>RF.CIUmid)], lty=2, col=gray(0.5), lwd=2)

xcoords<-ageseq[RFmid.index]

polygon(x=c(xcoords, rev(xcoords)),

y=c(RF.CILmid[RFmid.index], rev(RF.CIUmid[RFmid.index])),

col=adjustcolor(rural.col, alpha.f = 0.30),

border = NA)

xcoords<-ageseq[UFmid.index]

polygon(x=c(xcoords, rev(xcoords)),

y=c(UF.CILmid[UFmid.index], rev(UF.CIUmid[UFmid.index])),

col=adjustcolor(urban.col, alpha.f = 0.30),

border = NA)

###

plot(RMmid$HKDT~I(RMmid$Age + 0.1), type="p", pch=1, lwd=2, cex=2, col=rural.col,

xlim=range(adults$Age),

ylim=range(adults$HKDT),

xlab="",

ylab="",

cex.lab=1.5, cex.axis=1.3,

main="Mid-European males", cex.main=2)

points(UMmid$HKDT~I(UMmid$Age - 0.1), type="p", pch=1, lwd=2, cex=2, col=urban.col)

RMmid.index<-ageseq>=min(RMmid$Age) & ageseq<=max(RMmid$Age)

UMmid.index<-ageseq>=min(UMmid$Age) & ageseq<=max(UMmid$Age)

lines(RMmid.fit[RMmid.index]~ageseq[RMmid.index], lwd=3, lty=1, col=rural.col)

lines(UMmid.fit[UMmid.index]~ageseq[UMmid.index], lwd=3, lty=1, col=urban.col)

abline(v=ageseq[match(FALSE, UMmid.fit>RM.CIUmid)], lty=2, col=gray(0.5), lwd=2)

xcoords<-ageseq[RMmid.index]

polygon(x=c(xcoords, rev(xcoords)),

y=c(RM.CILmid[RMmid.index], rev(RM.CIUmid[RMmid.index])),

col=adjustcolor(rural.col, alpha.f = 0.30),

border = NA)

xcoords<-ageseq[UMmid.index]

polygon(x=c(xcoords, rev(xcoords)),

y=c(UM.CILmid[UMmid.index], rev(UM.CIUmid[UMmid.index])),

col=adjustcolor(urban.col, alpha.f = 0.30),

border = NA)

###

par(mar=c(1,1,1,1))

plot(1:2,1:2,type="n",bty="n",xaxt="n",yaxt="n")

mtext("Days since eclosion",side=1,line=-3,cex=1.5)

###Figure HKDT variation in young adults

index<-numeric(nrow(adults))

index[{adults$region=="Nordic" & adults$Age<=3}]<-1

index[{adults$region=="Central-European" & adults$sex=="female" & adults$Age<=3}]<-1

index[{adults$region=="Central-European" & adults$sex=="male" & adults$Age<=4}]<-1

adults.Y<-adults[index==1,]

###

Fmid<-subset(adults.Y, {sex=="female" & region=="Central-European"})

Mmid<-subset(adults.Y, {sex=="male" & region=="Central-European"})

Fnor<-subset(adults.Y, {sex=="female" & region=="Nordic"})

Mnor<-subset(adults.Y, {sex=="male" & region=="Nordic"})

###

ci<-function(x,cilevel=0.95){

m<-mean(x)

s<-sd(x)

n<-length(x)

df<-n-1

t<-qt(1-(1-cilevel)/2,df)

cil<-m-t*s/sqrt(n)

ciu<-m+t*s/sqrt(n)

output<-c(m,cil,ciu)

names(output)<-c("mean","lower","upper")

output

}

FMid.CI<-tapply(Fmid$HKDT, Fmid$environment, ci)

MMid.CI<-tapply(Mmid$HKDT, Mmid$environment, ci)

FNor.CI<-tapply(Fnor$HKDT, Fnor$environment, ci)

MNor.CI<-tapply(Mnor$HKDT, Mnor$environment, ci)

#####

rural.col<-"#E69F00"

urban.col<-"#0072B2"

mat=matrix(c(1, 2, 3), 1, 3, byrow=TRUE)

nf=layout(mat, respect=TRUE, widths=c(1, 3, 3),

heights=c(4))

layout.show(nf)

###

par(mar=c(1, 1, 1, 1))

plot(1:2, 1:2, type="n", bty="n", xaxt="n", yaxt="n")

mtext("Heat knock-down time (s)", side=2, line=-5, cex=1.5)

###

par(mar=c(4, 3, 2, 1))

plot(Fnor$HKDT~Fnor$Sex, type="n", pch=1, lty=0, cex=2, col="white",

ylim=range(adults$HKDT)-c(20, 0), range=0,

xlab="",

ylab="",

cex.lab=1.5, cex.axis=2,

main="North-European adults", cex.main=2)

xcoords<-runif(nrow(Fnor[Fnor$environment=="rural",]), min=0.75, max=0.85)

points(Fnor$HKDT[Fnor$environment=="rural"]~xcoords, pch=1, cex=1.5, col=rural.col)

arrows(0.8, FNor.CI$rural[1], 0.8, FNor.CI$rural[2], lwd=3, length=0.1, angle=90, col=1)

arrows(0.8, FNor.CI$rural[1], 0.8, FNor.CI$rural[3], lwd=3, length=0.1, angle=90, col=1)

points(0.8, FNor.CI$rural[1], pch=1, lwd=3, cex=2, col=1)

text(x=0.8, y=20, labels=paste("N=", nrow(Fnor[Fnor$environment=="rural",]), sep=""), cex=1.5)

xcoords<-runif(nrow(Fnor[Fnor$environment=="urban",]), min=1.15, max=1.25)

points(Fnor$HKDT[Fnor$environment=="urban"]~xcoords, pch=1, cex=1.5, col=urban.col)

arrows(1.2, FNor.CI$urban[1], 1.2, FNor.CI$urban[2], lwd=3, length=0.1, angle=90, col=1)

arrows(1.2, FNor.CI$urban[1], 1.2, FNor.CI$urban[3], lwd=3, length=0.1, angle=90, col=1)

points(1.2, FNor.CI$urban[1], pch=1, lwd=3, cex=2, col=1)

text(x=1.2, y=20, labels=paste("N=", nrow(Fnor[Fnor$environment=="urban",]), sep=""), cex=1.5)

xcoords<-runif(nrow(Mnor[Mnor$environment=="rural",]), min=1.75, max=1.85)

points(Mnor$HKDT[Mnor$environment=="rural"]~xcoords, pch=1, cex=1.5, col=rural.col)

arrows(1.8, MNor.CI$rural[1], 1.8, MNor.CI$rural[2], lwd=3, length=0.1, angle=90, col=1)

arrows(1.8, MNor.CI$rural[1], 1.8, MNor.CI$rural[3], lwd=3, length=0.1, angle=90, col=1)

points(1.8, MNor.CI$rural[1], pch=1, lwd=3, cex=2)

text(x=1.8, y=20, labels=paste("N=", nrow(Mnor[Mnor$environment=="rural",]), sep=""), cex=1.5)

xcoords<-runif(nrow(Mnor[Mnor$environment=="urban",]), min=2.15, max=2.25)

points(Mnor$HKDT[Mnor$environment=="urban"]~xcoords, pch=1, cex=1.5, col=urban.col)

arrows(2.2, MNor.CI$urban[1], 2.2, MNor.CI$urban[2], lwd=3, length=0.1, angle=90, col=1)

arrows(2.2, MNor.CI$urban[1], 2.2, MNor.CI$urban[3], lwd=3, length=0.1, angle=90, col=1)

points(2.2, MNor.CI$urban[1], pch=1, lwd=3, cex=2)

text(x=2.2, y=20, labels=paste("N=", nrow(Mnor[Mnor$environment=="urban",]), sep=""), cex=1.5)

legend("topleft", legend=c("rural", "urban"),

pch=16, col=c(rural.col, urban.col),

lwd=1, pt.cex=2, cex=2, lty=NA)

#########

plot(Fmid$HKDT~Fmid$Sex, type="n", pch=1, lty=0, cex=2, col="white",

ylim=range(adults$HKDT)-c(20, 0), range=0,

xlab="",

ylab="",

cex.lab=1.5, cex.axis=2,

main="Mid-European adults", cex.main=2)

xcoords<-runif(nrow(Fmid[Fmid$environment=="rural",]), min=0.75, max=0.85)

points(Fmid$HKDT[Fmid$environment=="rural"]~xcoords, pch=1, cex=1.5, col=rural.col)

arrows(0.8, FMid.CI$rural[1], 0.8, FMid.CI$rural[2], lwd=3, length=0.1, angle=90, col=1)

arrows(0.8, FMid.CI$rural[1], 0.8, FMid.CI$rural[3], lwd=3, length=0.1, angle=90, col=1)

points(0.8, FMid.CI$rural[1], pch=1, lwd=3, cex=2, col=1)

text(x=0.8, y=20, labels=paste("N=", nrow(Fmid[Fmid$environment=="rural",]), sep=""), cex=1.5)

xcoords<-runif(nrow(Fmid[Fmid$environment=="urban",]), min=1.15, max=1.25)

points(Fmid$HKDT[Fmid$environment=="urban"]~xcoords, pch=1, cex=1.5, col=urban.col)

arrows(1.2, FMid.CI$urban[1], 1.2, FMid.CI$urban[2], lwd=3, length=0.1, angle=90, col=1)

arrows(1.2, FMid.CI$urban[1], 1.2, FMid.CI$urban[3], lwd=3, length=0.1, angle=90, col=1)

points(1.2, FMid.CI$urban[1], pch=1, lwd=3, cex=2, col=1)

text(x=1.2, y=20, labels=paste("N=", nrow(Fmid[Fmid$environment=="urban",]), sep=""), cex=1.5)

xcoords<-runif(nrow(Mmid[Mmid$environment=="rural",]), min=1.75, max=1.85)

points(Mmid$HKDT[Mmid$environment=="rural"]~xcoords, pch=1, cex=1.5, col=rural.col)

arrows(1.8, MMid.CI$rural[1], 1.8, MMid.CI$rural[2], lwd=3, length=0.1, angle=90, col=1)

arrows(1.8, MMid.CI$rural[1], 1.8, MMid.CI$rural[3], lwd=3, length=0.1, angle=90, col=1)

points(1.8, MMid.CI$rural[1], pch=1, lwd=3, cex=2)

text(x=1.8, y=20, labels=paste("N=", nrow(Mmid[Mmid$environment=="rural",]), sep=""), cex=1.5)

xcoords<-runif(nrow(Mmid[Mmid$environment=="urban",]), min=2.15, max=2.25)

points(Mmid$HKDT[Mmid$environment=="urban"]~xcoords, pch=1, cex=1.5, col=urban.col)

arrows(2.2, MMid.CI$urban[1], 2.2, MMid.CI$urban[2], lwd=3, length=0.1, angle=90, col=1)

arrows(2.2, MMid.CI$urban[1], 2.2, MMid.CI$urban[3], lwd=3, length=0.1, angle=90, col=1)

points(2.2, MMid.CI$urban[1], pch=1, lwd=3, cex=2)

text(x=2.2, y=20, labels=paste("N=", nrow(Mmid[Mmid$environment=="urban",]), sep=""), cex=1.5)

#####

# The analysis without the Divci Hrady population was repeated with the above procedure

# but just replacing the data with a subset where the Divci Hrady population is excluded.

####################################################################################################

# Correlation between larval and adult HKDTs

# ------------------------------------------

# Use family means to avoid issues with within-family dependency of observations

L.adults<-adults[adults$Tested.as.larva=="yes",]

L.adults$family0<-as.character(L.adults$family)

L.adults$family<-factor(L.adults$family)

L.adults$ID<-paste(L.adults$family0, L.adults$Individual, sep=".")

str(L.adults)

table(table(L.adults$family))

A.larvae<-larvae[{larvae$ID %in% L.adults$ID},]

A.HKDTs<-tapply(L.adults$HKDT, L.adults$family, mean)

L.HKDTs<-tapply(A.larvae$HKDT, A.larvae$family, mean)

L.HKDTs<-L.HKDTs[names(L.HKDTs) %in% names(A.HKDTs)]

HKDTs<-data.frame(family=names(A.HKDTs), L.HKDT=L.HKDTs, A.HKDT=A.HKDTs)

# scatterplot

par(mar=c(5,5,2,2))

plot(HKDTs$A.HKDT~HKDTs$L.HKDT,

xlab="Larval HKDT (s)",

ylab="Adult HKDT (s)",

cex.lab=1.5, cex.axis=1.3,

pch=1, cex=2, lwd=2)

# correlation test

cor.test(HKDTs$A.HKDT, HKDTs$L.HKDT)

##############################################################################################

# 'Bootstrap' resampling to increase sample size for assessing sample size

# effect on larval results

# ------------------------------------------------------------------------

table(adults$environment, adults$region)

# Adult data includes 169 and 84 rural individuals from Mid-European and North-European

# regions, respectively, and 231 and 122 urban ones. These are the target

# sample sizes when resampling larval data.

ruralL.mid<-larvae[{larvae$environment=="rural" & larvae$region=="Central-European"},]

urbanL.mid<-larvae[{larvae$environment=="urban" & larvae$region=="Central-European"},]

ruralL.nor<-larvae[{larvae$environment=="rural" & larvae$region=="Nordic"},]

urbanL.nor<-larvae[{larvae$environment=="urban" & larvae$region=="Nordic"},]

indexR.mid<-1:nrow(ruralL.mid)

indexU.mid<-1:nrow(urbanL.mid)

indexR.nor<-1:nrow(ruralL.nor)

indexU.nor<-1:nrow(urbanL.nor)

# Number of bootstrap resamples

B<-5000

coefficients.avg<-matrix(nrow=B, ncol=4)

SEs.avg<-matrix(nrow=B, ncol=4)

avg.index<-numeric(B)

library(nlme)

library(MuMIn)

set.seed(123)

for(i in 1:B){

i.R.mid<-sample(x=indexR.mid, size=169, replace=TRUE)

i.U.mid<-sample(x=indexU.mid, size=231, replace=TRUE)

i.R.nor<-sample(x=indexR.nor, size=84, replace=TRUE)

i.U.nor<-sample(x=indexU.nor, size=122, replace=TRUE)

dataR.mid<-ruralL.mid[i.R.mid,]

dataU.mid<-urbanL.mid[i.U.mid,]

dataR.nor<-ruralL.nor[i.R.nor,]

dataU.nor<-urbanL.nor[i.U.nor,]

data<-rbind(dataR.mid, dataU.mid, dataR.nor, dataU.nor)

data$Cmass<-data$mass - mean(data$mass)

model<-lme(HKDT~Cmass*environment,

random=~1|family,

data=data,

method="ML",

weights=varIdent(form=~1|region))

models<-dredge(global.model=model, beta="none", rank="AICc")

models.avg<-get.models(models, subset = delta < 8)

{if (length(models.avg)==1){

coefficients.avg[i,]<-as.numeric(fixef(models.avg[[1]]))

SEs.avg[i,]<-coef(summary(models.avg[[1]]))[, "Std.Error"]

avg.index[i]<-1}

else {

avg.model<-model.avg(models.avg, fit=TRUE)

coefficients.avg[i,]<-avg.model$coefficients[1,]

SEs.avg[i,]<-summary(avg.model)$coefmat.full[, "Adjusted SE"]

}}

}

colnames(coefficients.avg)<-names(fixef(model))

colnames(SEs.avg)<-names(fixef(model))

zvalues.avg<-abs(coefficients.avg/SEs.avg)

table(avg.index)

###

# A figure illustrating the results

mat=matrix(c(0,0,1,2,3,4,6,7,8,9,5,6,10,11,12,0,0,13,13,13), 4, 5, byrow=TRUE)

nf=layout(mat, respect=TRUE, widths=c(2.5, 1, 4, 4, 4),

heights=c(1, 3, 3, 1))

layout.show(nf)

###

par(mar=c(1, 1, 1, 1))

plot(1:2, 1:2, type="n", bty="n", xaxt="n", yaxt="n")

mtext("Centered mass", side=3, line=-5, cex=1.3, font=2)

plot(1:2, 1:2, type="n", bty="n", xaxt="n", yaxt="n")

mtext("Environment (urban)", side=3, line=-5, cex=1.3, font=2)

plot(1:2, 1:2, type="n", bty="n", xaxt="n", yaxt="n")

mtext("Centered mass *

Environment (urban)", side=3, line=-5, cex=1.3, font=2)

plot(1:2, 1:2, type="n", bty="n", xaxt="n", yaxt="n")

mtext("Fixed effect

coefficient", side=3, line=-7, cex=1.3, font=2)

plot(1:2, 1:2, type="n", bty="n", xaxt="n", yaxt="n")

mtext("z-value", side=3, line=-7, cex=1.3, font=2)

plot(1:2, 1:2, type="n", bty="n", xaxt="n", yaxt="n")

mtext("Frequency", side=2, line=-3, cex=1.3)

###

par(mar=c(3, 3, 2, 1))

hist(coefficients.avg[,"Cmass"], breaks=40, xlab="", ylab="", main="", cex.axis=1.3)

CIs<-quantile(coefficients.avg[,"Cmass"], probs=c(0.025, 0.975))

abline(v=CIs, lwd=2, lty=2, col=gray(0.5))

abline(v=avg.model.l0b$coefficients[1, "Cmass"], col=2, lwd=3)

hist(coefficients.avg[,"environmenturban"], breaks=40, xlab="", ylab="", main="", cex.axis=1.3)

CIs<-quantile(coefficients.avg[,"environmenturban"], probs=c(0.025, 0.975))

abline(v=CIs, lwd=2, lty=2, col=gray(0.5))

abline(v=avg.model.l0b$coefficients[1, "environmenturban"], col=2, lwd=3)

hist(coefficients.avg[,"Cmass:environmenturban"], breaks=40, xlab="", ylab="", main="", cex.axis=1.3)

CIs<-quantile(coefficients.avg[,"Cmass:environmenturban"], probs=c(0.025, 0.975))

abline(v=CIs, lwd=2, lty=2, col=gray(0.5))

abline(v=avg.model.l0b$coefficients[1, "Cmass:environmenturban"], col=2, lwd=3)

hist(zvalues.avg[,"Cmass"], breaks=40, xlab="", ylab="", main="", cex.axis=1.3)

CIs<-quantile(zvalues.avg[,"Cmass"], probs=c(0.025, 0.975))

abline(v=CIs, lwd=2, lty=2, col=gray(0.5))

abline(v=summary(avg.model.l0b)$coefmat.full["Cmass", "z value"], col=2, lwd=3)

hist(zvalues.avg[,"environmenturban"], breaks=40, xlab="", ylab="", main="", cex.axis=1.3)

CIs<-quantile(zvalues.avg[,"environmenturban"], probs=c(0.025, 0.975))

abline(v=CIs, lwd=2, lty=2, col=gray(0.5))

abline(v=summary(avg.model.l0b)$coefmat.full["environmenturban", "z value"], col=2, lwd=3)

hist(zvalues.avg[,"Cmass:environmenturban"], breaks=40, xlab="", ylab="", main="", cex.axis=1.3)

CIs<-quantile(zvalues.avg[,"Cmass:environmenturban"], probs=c(0.025, 0.975))

abline(v=CIs, lwd=2, lty=2, col=gray(0.5))

abline(v=summary(avg.model.l0b)$coefmat.full["Cmass:environmenturban", "z value"], col=2, lwd=3)

###

par(mar=c(1, 1, 1, 1))

plot(1:2, 1:2, type="n", bty="n", xaxt="n", yaxt="n")

mtext("Value", side=1, line=-3, cex=1.3)
